# Hippocampal overexpression of NOS1AP promotes endophenotypes related to mental disorders

**DOI:** 10.1016/j.ebiom.2021.103565

**Published:** 2021-08-27

**Authors:** Florian Freudenberg, Esin Candemir, Xufeng Chen, Li-Li Li, Dilhan Esen-Sehir, Nicole Schenk, Makoto Kinoshita, Lena Grünewald, Veronika Frerichs, Nikolai Fattakhov, Jessica Manchen, Solmaz Bikas, Anita Kumar, Aet OLeary, David A. Slattery, Jakob von Engelhardt, Michael J. Courtney, Andreas Reif

**Affiliations:** a Department of Psychiatry, Psychosomatic Medicine and Psychotherapy, University Hospital, Goethe University, Frankfurt, Germany; b Graduate School of Life Sciences, University of Würzburg, Würzburg, Germany; c Institute of Pathophysiology, Mainz University Medical Center, Mainz, Germany; d Neuronal Signalling Laboratory, Turku Bioscience Centre, University of Turku and Åbo Akademi University, Turku, Finland; e The Faculty of Biological Sciences, Goethe University, Frankfurt, Germany

**Keywords:** NOS1AP, CAPON, nNOS, NOS-I, nitric oxide, psychiatric disorders

## Abstract

**Background:**

Nitric oxide synthase 1 adaptor protein (NOS1AP; previously named CAPON) is linked to the glutamatergic postsynaptic density through interaction with neuronal nitric oxide synthase (nNOS). NOS1AP and its interaction with nNOS have been associated with several mental disorders. Despite the high levels of NOS1AP expression in the hippocampus and the relevance of this brain region in glutamatergic signalling as well as mental disorders, a potential role of hippocampal NOS1AP in the pathophysiology of these disorders has not been investigated yet.

**Methods:**

To uncover the function of NOS1AP in hippocampus, we made use of recombinant adeno-associated viruses to overexpress murine full-length NOS1AP or the NOS1AP carboxyterminus in the hippocampus of mice. We investigated these mice for changes in gene expression, neuronal morphology, and relevant behavioural phenotypes.

**Findings:**

We found that hippocampal overexpression of NOS1AP markedly increased the interaction of nNOS with PSD-95, reduced dendritic spine density, and changed dendritic spine morphology at CA1 synapses. At the behavioural level, we observed an impairment in social memory and decreased spatial working memory capacity.

**Interpretation:**

Our data provide a mechanistic explanation for a highly selective and specific contribution of hippocampal NOS1AP and its interaction with the glutamatergic postsynaptic density to cross-disorder pathophysiology. Our findings allude to therapeutic relevance due to the druggability of this molecule.

**Funding:**

This study was funded in part by the DFG, the BMBF, the Academy of Finland, the NIH, the Japanese Society of Clinical Neuropsychopharmacology, the Ministry of Education of the Russian Federation, and the European Community.

**Research in Context**

**Evidence before the study**

Nitric oxide synthase 1 adaptor protein (NOS1AP) has been suggested to inhibit neuronal nitric oxide synthase (nNOS) interaction with the glutamatergic postsynaptic density but has later been described as a functional mediator of nNOS. This system, and NOS1AP in particular, has been linked to different mental disorders, including schizophrenia, depression, and posttraumatic stress disorder. For example, genetic variants of NOS1AP have been associated with multiple mental disorder phenotypes and higher cortical and hippocampal expression was observed in patients with schizophrenia and depression. Underscoring these findings, we revealed that NOS1AP overexpression in cultured neurons disturbed growth of dendrites and reduced the number of dendritic spines, similar to what is observed in patients of different mental conditions. Thus, NOS1AP represents an attractive novel target for psychiatric disorders.

**Added value of the study**

This is the first study to assess how increased NOS1AP expression in the hippocampus affects mental disorder related phenotypes. Using viral gene transfer we found that overexpression of murine NOS1AP in the dorsal hippocampus of wild type mice leads to deficits in social memory and working memory capacity, i.e. behavioural phenotypes linked to different mental conditions. Other cross-disorder phenotypes such as anxiety, startle, prepulse inhibition of the startle response, anhedonia, and spatial reference memory, were not affected, implying a more specific association of hippocampal NOS1AP in mental disorder-related behaviours. Morphologically, we found that hippocampal NOS1AP overexpression reduced the dendritic spine density akin to observations in post-mortem samples from patients. This lends further weight to our previous findings in cultured neurons. Molecular analysis showed that NOS1AP overexpression increased the interaction of nNOS with the postsynaptic density without affecting the expression of interaction partners. Thus, we provide further evidence that NOS1AP acts a mediator and not an inhibitor of nNOS.

**Implications of all the available evidence**

Our study suggests a link between genetically driven increased hippocampal NOS1AP expression to selective phenotypes of mental disorders that are suggestive of crossing classical disease boundaries. As NOS1AP and its protein interactions can be targeted using novel small molecule compounds our work provides an important contribution to potential novel treatment strategies in the context of precision medicine approaches.

Alt-text: Unlabelled box

## 1 Introduction

Nitric oxide synthase 1 adaptor protein (NOS1AP; previously named CAPON [Carboxy-terminal PDZ ligand of neuronal nitric oxide synthase]) is a scaffolding protein that has been linked to different mental disorders (reviewed in [1,2]). For example, elevated *NOS1AP* mRNA and protein was found in blood [3] and the dorsolateral prefrontal cortex (DLPFC) of patients with schizophrenia [4,5]. Increased NOS1AP immunoreactivity in the DLPFC and the anterior cingulate cortex, was described in patients with major depressive disorder [6]. Recently we found increased *NOS1AP* mRNA in hippocampus of schizophrenia patients (in preparation). Consistent with these changes in expression, *NOS1AP* variants have been associated with schizophrenia endophenotypes [7], [8], [9] and depression-related traits in schizophrenia patients [10]. Moreover, *NOS1AP* variants have been associated with symptom severity, and depression and anxiety symptoms in posttraumatic stress disorder (PTSD) [11,12].

While it is unclear whether increased NOS1AP expression directly contributes to psychopathology or endophenotypes, several preclinical studies hint at such a possibility. Overexpression of murine NOS1AP in the mouse dentate gyrus had anxiogenic effects [13] and downregulation of NOS1AP in the medial prefrontal cortex reversed stress-induced depression-like behaviour in mice [6]. Moreover, NOS1AP overexpression in cultured neurons reduced the dendritic growth and the number of mature dendritic spines, and increased filopodia-like protrusions [14], [15], [16], [17], [18]. These findings are akin to observations in post-mortem studies of different mental conditions including schizophrenia, mood disorders, and intellectual disability (e.g. reviewed in [19,20]).

NOS1AP is best known for its interaction with the PDZ domain of neuronal nitric oxide synthase (nNOS or NOS-I, encoded by the *NOS1* gene) through an internal ExF motif [21] and a PDZ-motif [22], both contained in the carboxyterminus of full-length NOS1AP (i.e. amino acids 400-506 in humans). In neurons, nNOS is linked to the postsynaptic density (PSD) of glutamatergic synapses [23,24] through interaction with the PDZ2 domain of PSD-93 or -95 [25], [26], [27], [28]. This interaction brings nNOS in proximity to NMDA receptors enabling NMDA receptor-dependent Ca^2+^ influx to activate nNOS [29,30] resulting in further downstream effects that are involved in neuronal plasticity.

NOS1AP was originally described as an inhibitor of nNOS/PSD-95 interaction [22]. However, subsequent findings showed NOS1AP mediated NMDA receptor signalling through nNOS [31], [32], [33], which depends on nNOS/PSD-95 interaction [30], suggesting that NOS1AP may in fact mediate, not inhibit the function of the nNOS/PSD-95/NMDA receptor complex. Models have been proposed to reconcile these apparently conflicting findings (discussed in [34]). In addition to nNOS, NOS1AP interacts with other proteins, including RasD1 (also known as DexRas1; encoded by *RASD1*) [31] and MKK3 (encoded by *MAP2K3*) [33], linking these proteins to nNOS and, thereby, mediating their activation (reviewed in [2,34]).

Together, these findings strongly argue that NOS1AP is a key effector component of glutamatergic pathways, and in doing so, it may have a role in psychiatric phenotypes across diagnostic boundaries. This is in good agreement with the current understanding about the involvement of the glutamatergic system in the pathophysiology of several mental disorders (see e.g. [35], [36], [37], [38]) as also suggested by cross-disorder genetics [39,40].

The hippocampus, a highly interconnected glutamatergic brain region, has been suggested as an important brain structure for mental disorders and related phenotypes (see e.g. [41], [42], [43], [44], [45]) as also supported by cross-disorder brain imaging studies [46]. Despite the important role of nitric oxide signalling in the hippocampus [47] and the above-described increase of *NOS1AP* mRNA in patients suffering from schizophrenia, a potential involvement of hippocampal NOS1AP and its interaction with the nNOS/PSD-95/NMDA receptor complex to different endophenotypes of severe mental disorders has not been investigated yet. To further clarify the neural and behavioural circuits that are affected by NOS1AP and thus to disentangle the psychiatric phenotypes linked to disturbed NMDA/nNOS/NOS1AP signalling, we overexpressed murine NOS1AP or its carboxyterminal tail (i.e. NOS1AP_396-503_) required for NOS1AP/nNOS interaction [21] in the hippocampus of wild type mice and studied the resulting changes in gene expression, neuronal morphology, and behaviour.

## 2 Methods

### 2.1 Viral vectors

Cloning of the pAAV plasmids coding for mCherry (pAAV-hSyn-mCherry.3xFLAG-WPRE; RRID:Addgene_127861) and murine NOS1AP (NM_001109985; pAAV-hSyn-mCherry.3xFLAG.NOS1AP-WPRE; RRID:Addgene_127864), was previously described [14]. The pAAV-hSyn-mCherry.3xFLAG.NOS1AP_396-503_-WPRE (RRID:Addgene_174133) plasmid was cloned analogous to the NOS1AP plasmid [14]. In short, the DNA sequence coding for the carboxyterminal 108 amino acids of murine NOS1AP (NM_001109985) was amplified from mouse cDNA using primers NOS1AP_396-503_-F and NOS1AP_396-503_-R (Table S1). The resulting PCR product was inserted in frame into the NheI and HindIII sites of pAAV-hSyn-mCherry.3xFLAG-WPRE) resulting in pAAV-hSyn-mCherry.3xFLAG.NOS1AP_396-503_-WPRE.

All constructs contained the human Synapsin-1 promoter, limiting expression to neurons [48]. Recombinant adeno-associated viruses (rAAVs) were generated and titrated to 2 × 10^9^ viral genomes/µl using WPRE-specific primers (Table S1) as described [14].

All virus plasmids are available from Addgene (https://www.addgene.org/Florian_Freudenberg/).

### 2.2 Mice

Wild-type male C57BL/6JRj mice (Janvier Labs; RRID:MGI:2670020) were maintained under controlled conditions (lights on 0700-1900; 21±1 °C; 55±5% humidity) with ad libitum access to food and water. Mice were tested during the light-phase by evaluators blinded to the treatment. Mice were habituated to the experimental room >45 min before testing and exposed to 60 dB white noise while staying in the experimental room.

### 2.3 Ethics

All experiments were conducted according to the Council Directive 86/609/EEC of 24 November 1986 and German animal welfare laws (TierSchG and TSchV) and were approved by the Regierungspräsidium Darmstadt (approval ID: FK/1033).

### 2.4 Stereotaxic surgeries

Mice were randomly assigned to treatment groups (i.e. mCherry [control], NOS1AP, or NOS1AP_396-503_), with one mouse of each treatment group in a cage to exclude potential changes in social behaviour to unequally confound treatment groups. Mice were stereotaxically injected with the rAAVs in the dorsal hippocampus as described [49]. Briefly, mice were given metamizole (Ratiopharm GmbH) in the drinking water (2 mg/ml) 48 h before and after surgery. Surgeries were performed at 7 weeks of age using a stereotaxic frame (Stoelting Co) under 1-2% isoflurane anaesthesia (induction of anaesthesia at 5-6%). To avoid heat-loss during anaesthesia, the body temperature was maintained at 37 °C by a thermostatically controlled pad. For local anaesthesia, 100-200 µl ropivacaine (2 mg/ml) was administered subcutaneously 5-10 min before making an incision above the scalp. Small holes were drilled using 0·6 mm carbide burrs at stereotaxic coordinates for the dorsal hippocampus (AP: 2·1 mm, ML: ±1·6 mm, DV: 1·3 mm and 2·0 mm; 1 µl/injection site; flow rate: 0·1 µl/min) [49], [50], [51]. Mice were injected bilaterally with virus-containing solution using stereotaxic coordinates. Mice were closely monitored after surgery and were allowed to recover for 4-5 weeks before starting experiments. Before the start of the experiments, experimenters were blinded to the treatments and only unblinded after analyses were concluded.

### 2.5 Molecular analyses of mouse brain tissue

#### 2.5.1 Quantitative PCR (qPCR)

Hippocampal tissue from dorsal hippocampus of mice with virus-mediated overexpression of mCherry, NOS1AP, and NOS1AP_396-503_ in dorsal hippocampus (5 mice/group) was dissected 4-5 weeks after injection and stored in RNA preserving solution (25 mM sodium citrate, 10 mM EDTA, 5·3 M ammonium sulphate, pH 5·2) at 4 °C for one week. Hippocampi from both hemispheres were isolated on a cooling plate at 4 °C and then stored at -20 °C. RNA was isolated separately from all hippocampi (i.e. 10 hippocampi from 5 mice/group) using the MagJET RNA Kit (ThermoFisher Scientific) according to the manufacturer's instructions using a pipetting robot (Biomek NX^P^, Beckman Coulter). A total of 375 ng RNA per sample was reverse transcribed using the iScript™ cDNA Synthesis Kit (Bio-Rad), using both random hexamers and oligo(dT) primers (note that one NOS1AP_396-503_ sample could not be reverse transcribed due to too low RNA yield).

Target-specific quantitative PCR (qPCR) for *Actb, B2m, Hprt*, and *Sdha* (as reference genes) and for 17 NOS1AP-associated genes (i.e. genes of gene products directly interacting with NOS1AP or functionally dependent on NOS1AP interactions) *Cpe, Dlg1, Dlg3, Dlg4, Gria1, Gria2, Grin2a, Grin2b, Gucy1a1, Gucy1a2, Gucy1b1, Gucy1b2, Map2k3, Mapk14, Nos1, Nos1ap, Rasd1, Scrib*, and *Syn1* (as target genes) was performed in 10 µl reactions containing 5 µl AMPLIFYME SG No-ROX Mix (Blirt), target specific forward and reverse primers (final concentration: 0·3 µM each; see Table S2 for primer sequences), and cDNA (final dilution: 1:200) using a 384-well plate (primaPLATE, Steinbrenner Laborsysteme GmbH) with LightCycler® 480 Sealing Foil (Roche) on the LightCycler® 480 Instrument II (Roche). All reactions were run in duplicates. PCR conditions: 3 min at 95 °C, followed by 45 cycles of 5 s at 95 °C, 10 s at 60 °C, and 10 s at 72 °C followed by a plate read. At the end of amplification, a melting curve was generated. Crossing point (Cp) values were calculated by the LightCycler 480 software (release 1.5.1.62) using the Second Derivative Maximum method. Relative gene expression levels were analysed using GenEx6 v3.1.3 (MultiD Analyses AB). One of the NOS1AP_396-503_ samples was removed from further analysis as no reference gene data were available. For every target, a standard curve was created on the same plate as the target samples and Cp values were corrected for efficiencies calculated from these standard curves (see Table S2). Missing data points (*Hprt* [1 from mCherry and 2 from NOS1AP], *Dlg3* [1 from mCherry, 1 from NOS1AP], *Scrib* [1 from NOS1AP], *Gucy1a2* [1 from mCherry]) were imputed. Robustness of imputed data was confirmed by complete case analysis (i.e. reanalysis with imputed values removed; data not shown).

Expression data were calculated relative to the expression of *Sdha* which was selected as the most stable of the four reference genes by analysis with Normfinder. Data were calculated relative to the average of the mCherry group and converted to log2.

#### 2.5.2 Protein isolation and co-immunoprecipitation (co-IP)

Hippocampal tissue from dorsal hippocampus of uninjected mice, and from mice with virus-mediated overexpression of mCherry, NOS1AP, and NOS1AP_396-503_ (3 mice/group, 1 hippocampus/mouse) in dorsal hippocampus was dissected 4-5 weeks after injection and snap-frozen in isopentane chilled with dry ice. Frozen tissue was weighed, and homogenized while thawing in 30 µl/mg low-salt buffer (LSB [52]) supplemented with protease inhibitors, 1 mM DTT and nonionic detergent (0·5% Igepal CA-630) using 10 strokes of a Dounce homogenizer, and precleared at 20,000 x g/4 °C for 10 min. Protein content of each homogenate was quantified with the Bio-Rad DC protein assay kit (Bio-Rad) and equal amounts from each sample were used in parallel in the subsequent steps.

Co-immunoprecipitation (Co-IP) was performed as previously described [33]. Briefly, immunoprecipitating (IP) antibody, nNOS antibody (mouse monoclonal clone A-11, RRID:AB_626757, Santa Cruz Biotechnology, 2·5 μg/ml) was added to each lysate. Lysates were rotated for 2 h/4 °C, 5 μl of protein-A resin (GenScript) was added, and rotation continued for 1 h. The resin was washed three times with LSB, and protein was eluted from drained resin by incubation at 95 °C for 10 min in SDS-PAGE sample loading buffer and analysed by Western blotting. Immunoprecipitated nNOS, and co-immunoprecipitated NOS1AP and PSD-95 in all samples were determined by Western blotting with anti-nNOS antibody (rabbit polyclonal IgG, RRID:AB_2313734, Invitrogen), anti-NOS1AP antibody (rabbit polyclonal IgG, R-300, RRID:AB_2251417, Santa Cruz Biotechnology) and anti-PSD-95 antibody (mouse monoclonal IgG2a, clone K28/43, RRID:AB_2307331,UC Davis/NIH NeuroMab Facility) respectively. Secondary antibodies conjugated to horseradish peroxidase (Santa Cruz Biotechnology) were used and detected with enhanced chemiluminescence reagent (Thermo Scientific).

Immunoblots of co-IP and input protein were quantified using ImageJ. For analysis of the endogenous NOS1AP levels in the input samples, differences in intensities of the bands per lane required a more precise quantification as follows: Data from scanned exposures of blots trimmed to remove the bulk of excess signal from over-expressed protein (Fig. S2E) were collected from equal width regions of interest in ImageJ and imported to Excel. The data for each lane was fitted to Gaussians (one per band present in the analysed lane data) using the Excel solver add-in.

### 2.6 Dendritic spine analysis

One month after rAAV delivery to the dorsal hippocampus (5 mice/group), mice were killed by cervical dislocation, and brains were removed. The freshly dissected brains were fixed in 4% Histofix (Carl Roth) for 4 h at room temperature and then transferred to 30% sucrose in 1xPBS at 4 °C overnight. Brains were stained using the FD Rapid GolgiStain kit (FD NeuroTechnologies) using a modified procedure. Specifically, brains were immersed in FD Solution A + B at room temperature for 10 days followed by FD Solution C for 4 days. Brains were stored at -20 °C until sagittal sections (100 µm thickness) were made at -22 °C using a cryostat (Leica CM 3050 S). Sections were transferred to glass slides (Superfrost, ThermoFisher Scientific). After drying sections at room temperature for at least 24 h, slides were temporarily coverslipped with 20% Glycerol in 1xPBS and imaged for mCherry fluorescence at 2·5x (0·085 numerical aperture) on an Axio Observer.Z1 equipped with an Axiocam 506 mono and Colibri.2 LED light source (all microscopic equipment from ZEISS).

After imaging, coverslips were removed, slides were rinsed with double distilled water 2 × 3 min, and placed in a mixture containing FD Solution D and E (25% each) and double distilled water (50%) for 10 min. Brains were rinsed 2 × 3 min in double distilled water, dehydrated in a series of 50%, 75%, 95%, 100% ethanol (3 min each), and cleared in xylenes (2 × 3 min). Slices were coverslipped using Histofluid (Paul Marienfeld).

Golgi-stained dendrites were imaged using an EC Plan-Neofluar 40x (1·3 numerical aperture) Oil M27 objective combined with 1·6x Tubelens Optovar on an Axio Observer.Z1 equipped with an Axiocam 506 mono (all microscopic equipment from ZEISS). Z-stacks (∼20-30 µm Z-axis in total; distance between optical sections = 0·33 µm) were taken from randomly chosen secondary dendrites in the stratum radiatum of dorsal hippocampal CA1 pyramidal cells. For each viral vector, 25 secondary dendrites from 5 different mice (i.e. 5 dendrites/mouse) were analysed

Dendritic spines were analysed using the trainable classifier in NeuronStudio (version 0.9.92). Output classes were defined as stubby, mushroom, long-thin, thin, filopodia and branched. The training set contained 200 spines (24 stubby, 60 mushroom, 23 thin, 56 long-thin, 35 filopodia, 2 branched). A region of interest with a length of 30 µm was set and spines were classified according to the classification scheme. After automatic detection spine identification was manually verified and corrected. For analysis, thin and long-thin spines were combined. The total number of mature spines was calculated as the sum of all spine types excluding filopodia.

### 2.7 Analysis of behaviour

One month after rAAV delivery to the dorsal hippocampus (15 mice/group) the same set of mice were tested behaviourally for anxiety in the light dark box and the elevated zero maze, for locomotor activity in the open field, for sensorimotor gating by measuring the prepulse inhibition (PPI) of the acoustic startle response (ASR), for anhedonia by measuring sucrose preference, for spatial working memory (SWM) in the rewarded alternation task on the T-maze and the spatial novelty preference paradigm in the Y-maze, and for spatial reference memory in the Y-maze.

#### 2.7.1 Light dark box

The apparatus (Stoelting Co) consisted of a chamber made from infrared transparent black acrylic glass (W: 40 cm x L: 40 cm x H: 35 cm), separated into a brightly lit (∼400 lux) and a dark (∼3 lux illuminated by an infrared light source) compartment (each 20 × 40 cm in size) connected by an opening. Each mouse was placed in the dark compartment facing a corner and allowed to freely explore the light dark box for 10 min. Behaviour was recorded with an infrared sensitive USB camera (The Imaging Source) and the first 5 min were automatically quantified using ANY-maze (Stoelting Co).

#### 2.72 Elevated zero maze

The apparatus (Stoelting Co) consisted of a circular platform (diameter: 50 cm, lane width: 5 cm, elevated 50 cm from the ground) that contained two opposing closed sections (wall height: 15 cm; illumination: ∼30 lux) and two opposing open sections with a 5 mm high lip (illumination: ∼120 lux). Mice were placed on the start of a closed compartment facing that compartment and their behaviour was recorded under infrared illumination for 10 min by use of an infrared sensitive USB camera (The Imaging Source) and the first 5 min were automatically quantified using ANY-maze (Stoelting Co). One NOS1AP_396-503_ mouse fell off the maze after ∼1 min and was excluded from analysis.

#### 2.7.3 Open field

The apparatus (Stoelting Co) was surrounded by walls made from black acrylic glass (W: 40 cm x L: 40 cm x H: 35 cm). Each mouse was placed into the open field facing one of the corners and allowed to explore the chamber for 10 min. Horizontal activity was monitored using a USB camera (The Imaging Source) and vertical activity was measured using an infrared array (Stoelting Co). Behaviour was automatically quantified using ANY-maze (Stoelting Co).

#### 2.7.4 Social interaction and social recognition

One day after the open field test mice were placed back into the same arena (light level: 40 lux) for 1 min to re-habituate to the environment. A juvenile conspecific stimulus mouse (4 weeks of age) was placed into the open field and both mice could freely explore each other for 5 min. The test was terminated prematurely if the test mouse attacked the stimulus mouse. Mice were returned to their home cage and after 30 min the test mouse was returned to the open field and habituated again for 1 min. The same ‘familiar’ juvenile mouse and a ‘novel’ juvenile stimulus mouse were placed into the arena and they were allowed to freely explore each other for 5 min.

For both sessions, the first 3 min of interaction were manually evaluated using the ANY-maze software. Social interaction was defined as time spent when test mice explored (e.g., sniffing, touching, licking) the stimulus mice, i.e. investigation of the test mouse by the juvenile was not included in the scoring. All mice that attacked the juvenile interaction partner (1 mCherry and 1 NOS1AP expressing mouse each) and mice with barbered whiskers (2 mCherry, 1 NOS1AP, 1 NOS1AP_396-503_) were excluded from data analysis for social interaction.

#### 2.7.5 Prepulse inhibition (PPI) of the acoustic startle response (ASR)

PPI of the ASR was measured using the SR-LAB™ startle response system (San Diego Instruments, Inc., USA) as described previously [53]. Briefly, after 5 min acclimation to the background noise (65 dB white noise), mice were exposed to six startle pulse trials (120 dB broadband noise for 40 ms, 10 s inter trial interval [ITI]). Subsequently, mice were presented with 10 x no-stimulus, 10 x startle pulse, 10 x each prepulse (4, 8, 12, 16 dB above background = 69, 73, 77, 81 dB for 20 ms) followed after 80 ms by a startle pulse, 10 x prepulse only (81 dB) in pseudorandomized order with a variable ITI (20-30 s). The test session ended with six startle pulse trials separated by 10 s ITIs. Overall, this protocol lasted about 35 min. The magnitude of the ASR (whole body reflex) to pulse only trials were averaged for each mouse and defined as startle amplitude. PPI percentage was calculated using a custom written MATLAB script as described in [53] using the following formula:

$$\mathrm{PPI}\left(\%\right)=100\times \frac{\text{peak}1\;\text{startle}\;\text{amplitud}e_{\text{startle}\;\text{trials}}-\text{peak}1\text{startle}\;\text{amplitud}e_{\text{prepulse}+\text{startle}\;\text{trials}}}{\text{peak}\;1\;\text{startle}\;\text{amplitud}e_{\text{startle}\;\text{trials}}}$$

#### 2.7.8 Sucrose preference

To measure preference for a sucrose containing solution mice were provided with two bottles, one filled with 1% sucrose in tap water and another only containing tap water for 48 hours. To avoid any side preferences, bottles containing sucrose were first positioned on the right side of the feeder grid where mice were used to receive drinking water and bottles containing water were positioned to left of the grid. After 24 h, bottle positions were switched. Sucrose preference was calculated by the following formula:

$$Sucrose\;preference\left(\%\right)=100\times \frac{\text{Amount}\;\text{of}\;\text{sucrose}\;\text{solution}\;\text{consumed}}{\text{Total}\;\text{amount}\;\text{of}\;\text{liquid}\;\text{consumed}}$$

#### 2.7.9 Rewarded alternation task

Rewarded alternation was performed on a grey T-maze apparatus (Stoelting Co) with a start arm (35 cm x 5 cm) and two equal goal arms (28 cm x 5 cm) enclosed by 10 cm high walls as described previously [49,50]. In brief, mice were weighed daily and fed with chopped food pellets sufficient to maintain their body weight at 80-85% of their free-feeding weight. For each trial, 20 μl of sweetened condensed milk (Dovgan, 50% diluted in water) per goal arm was provided as a reward. A trial started by placing a mouse into the start arm facing away from the goal arms. Mice were forced into one of the goal arms by blocking the other goal arm and were retrieved after consuming the milk. Mice were placed back to the start arm, the block was removed, and they were allowed to freely choose one of the goal arms. The correct choice (previously unvisited arm) was rewarded. Animals received one session (4 trials) per day for the first two days and two sessions (4 trials each) per day for 11 days (i.e., 96 trials in total). Each mouse received an equal number of right and left arm runs. Performance was calculated as the percentage of correct choices per block (24 trials/block).

#### 2.7.10 Spatial novelty preference

Spatial novelty preference was performed as described [49] on a Y-maze apparatus (Stoelting Co) consisting of three equal arms (35 cm x 5 cm enclosed with 10 cm high walls). Briefly, for each mouse, one of the arms was randomly designated as the novel arm, which remained blocked, except during the last trial. The mouse was placed at the end of one of the unblocked arms (start arm) and allowed to freely explore both arms for 2 min after leaving the start arm. This procedure was repeated five times in total (i.e. 5 × 2 min trials) with 1 min ITIs and alternating the start arm. After the 5^th^ trial the block was removed from the novel arm and the mouse was placed back to the start arm to explore the maze for a final 2 min. Time spent exploring the novel arm was recorded using ANY-maze (Stoelting Co.) and preference for the novel arm was calculated.

#### 2.7.11 Spatial reference memory

Spatial reference memory was performed under food deprivation as described above (see Rewarded alternation task) using the same Y-maze as described for the spatial novelty preference paradigm. Each mouse was randomly assigned a goal arm that remained constant throughout training. For each trial, a mouse was placed into one of the other arms, which alternated pseudo-randomly. If a mouse chose the correct goal arm it received a reward (20 µl of sweetened condensed-milk, Dovgan, 50% diluted in water). Animals received two sessions (4 trials each) per day for the first two days and 3 sessions (4 trials each) for the following four days. Performance was calculated as the percentage of correct choices per block (16 trials/block).

### 2.8 Analysis of brains after behavioural analysis

Within two weeks after behavioural testing, animals were deeply anesthetized with 5-6% isoflurane and immediately fixed via transcardial perfusion with 6 ml 1x PBS solution followed by 30 ml 4% formaldehyde (FA) in 1x PBS with a perfusion speed of 3 ml/min. Brains were post-fixed in 4% FA in 1x PBS for 2 h, cryoprotected in 30% sucrose in 1x PBS for 48 h and then frozen at -20 °C. Coronal sections (20 µm thickness) were made at -20 °C using a Leica CM 3050 S cryostat and transferred to glass slides (Superfrost, ThermoFisher Scientific). Sections were kept at -80 °C until further use.

Glass slides with brain sections were mounted with a coverslip using Fluoroshield mounting medium (Sigma-Aldrich) and analysed at 2·5x magnification on a fluorescence microscope Axio Observer Z1 (ZEISS). Viral infections were assessed in CA1/CA2, CA3 and dentate gyrus (DG) separately by applying a semi-quantitative value on a scale from 0 to 4 (0 = no mCherry fluorescence visible, 1 = ∼1-25% cells showing mCherry fluorescence, 2 = ∼25-50%, 3 = ∼50-75%, 4 = ∼75-100%). Slices between ∼-1·3 mm to ∼-2·7 mm from bregma according to the mouse brain atlas [54] with 200 µm between sections were scored for both hemispheres and the results were averaged to obtain a mean score for the dorsal hippocampus. One mCherry mouse could not be analysed, as it died prematurely of natural causes and the brain was not recovered.

### 2.9 Statistical analyses

Data that passed criteria for homogeneity of variances (Levene's test P>0·05) and normal distribution (Shapiro-Wilk tests P>0·05) were analysed by analysis of variance (ANOVA). Post-hoc analysis was performed using Fisher's least significant difference (LSD) method (i.e. uncorrected t-tests using a pooled error term), which in the case of three treatment groups (as is the case in this study) retains the alpha level (probability for type I error) at the nominal level (here 5%), while providing increased statistical power when compared to other methods [55], [56], [57].

If the ANOVA criteria for variance homogeneity and/or normality were violated (i.e. Levene's test and/or Shapiro-Wilk test P≤0·05), non-parametric tests (Kruskal-Wallis test for between-group comparisons and Friedman test or, if only two groups were compared, Wilcoxon signed-rank test for within-group comparisons) were used. Dunn's test (uncorrected, analogous to the LSD) was used for post-hoc comparison.

Specifically, co-IP, open field, light-dark-box, elevated zero maze, startle, and spatial novelty preference data all met homogeneity and normality criteria and were analysed by one-way ANOVA. Startle habituation data also met these criteria and was analysed by repeated measures ANOVA. The data for mCherry fluorescence, dendritic spines, sucrose preference, and social interaction violated heterogeneity and/or normality criteria and were analysed using the Kruskal-Wallis test. Data for social recognition, rewarded alternation, and reference memory violated at least one of the criteria and thus between-group comparisons were performed using the Kruskal-Wallis test, and within group comparisons were performed using the Wilcoxon signed-rank test. PPI data violated the normality criterium and within-group comparisons across prepulse intensities were performed using Friedman tests for each group, while between-group comparison were performed using Kruskal-Wallis tests for each prepulse intensity.

For the qPCR, some of the targets violated homogeneity and/or normality criteria and thus all qPCR data were analysed using the Kruskal-Wallis test. The significant P-value (i.e. significance threshold) was Bonferroni-corrected for the number of tested targets (P_Bonf_=$\frac{0\cdot 05}{17\;\text{target}\;\text{genes}}\;$=0·00294117).

To analyse sensitivity of our findings we have repeated all statistical analyses, but with outliers (defined as values that exceed 1.5 x interquartile range below or above the 1^st^ or 3^rd^ quartile respectively) removed. Any outcomes that changed as a result of this analysis are reported accordingly.

For statistical analysis we used JASP (version 0.14.1) and Jamovi (version 2.0.0.0).

Sample sizes required to achieve 80% or more power at an alpha level of 5% were calculated a priori and achieved power was confirmed post-hoc for all experiments using G*Power (version 3.1.9.7) [58,59].

### 2.10 Role of the funding source

The funding agencies had no further role in study design, in the collection, analysis and interpretation of data, in the writing of the report and in the decision to submit the paper for publication.

## 3 Results

### 3.1 Hippocampal overexpression of NOS1AP increases the nNOS/PSD-95 interaction

**Fig. 1:**
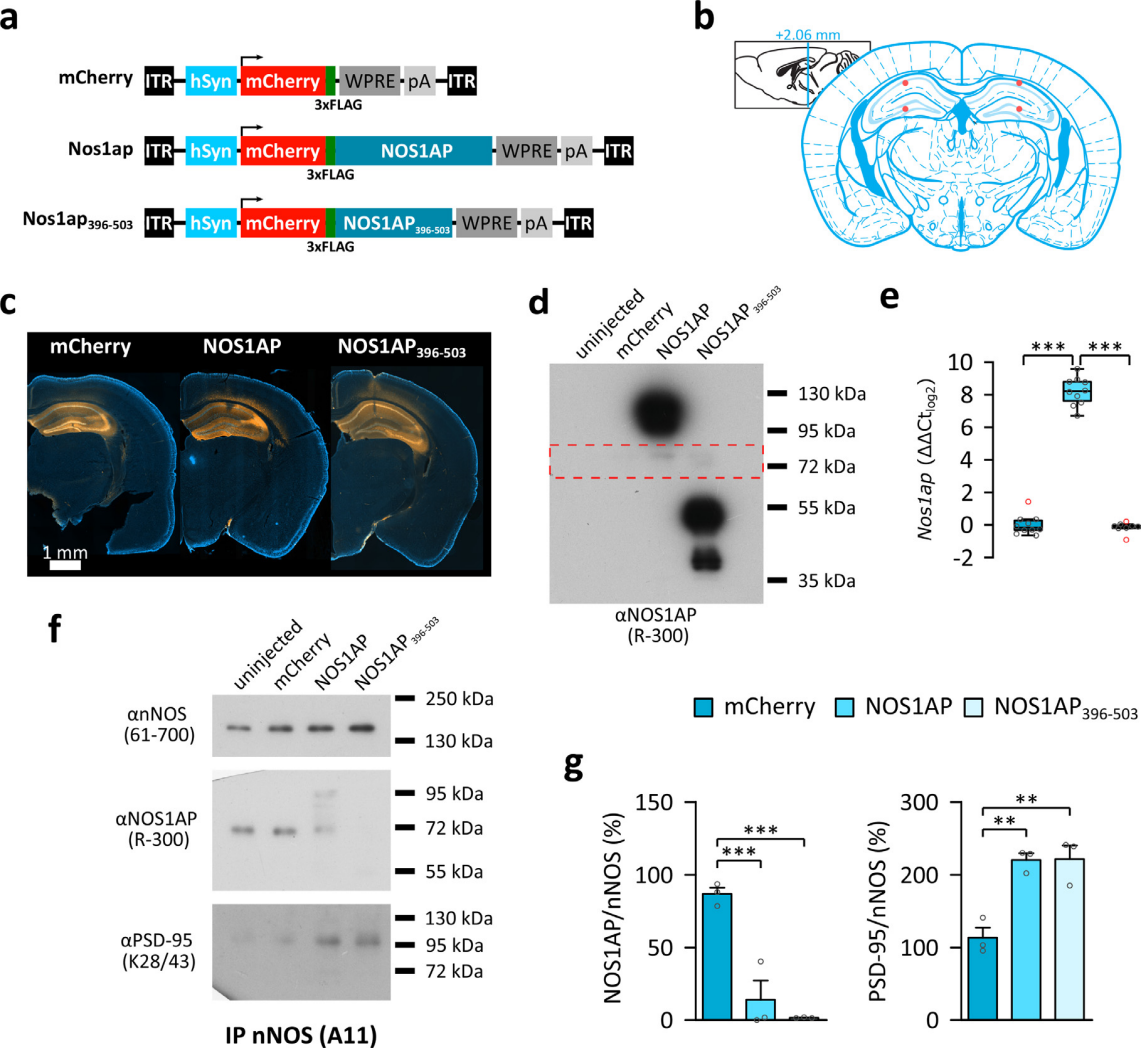
Hippocampus-specific overexpression of NOS1AP and NOS1AP_396-503_ enhances the interaction between PSD-95 and nNOS. (a) Schematic representation of the recombinant adeno-associated virus (rAAV) vectors used for expression of mCherry, the full-length long isoform of NOS1AP, and the carboxyterminal nNOS interaction domain of NOS1AP (i.e. NOS1AP_396-503_). (b) Schematic edited from [54] depicting the injection sites in dorsal hippocampus (red dots). (c) Example images showing mCherry fluorescence in dorsal hippocampus of mice injected with the mCherry, NOS1AP, and NOS1AP_396-503_ expressing rAAVs. (d) Example immunoblot of protein lysates from dorsal hippocampus of C57BL/6JRj mice stained with an antibody raised against the carboxyterminal domain of NOS1AP. Endogenous NOS1AP is faintly visible at ∼72 kDa (indicated by the red box; for semi-quantitative analysis see Fig. S2). Overexpression of the virally encoded NOS1AP and NOS1AP_396-503_ containing the mCherry and 3XFLAG tag can be seen at ∼95 kDa and ∼50 kDa respectively but could not be quantified as the signal differences are beyond the linear range of the film. (e) Quantification of *Nos1ap* mRNA using quantitative PCR (qPCR). Data were normalized to the expression of *Sdha* and calculated relative to the average expression of the mCherry injected group (N=10 hippocampi from 5 mice). Data are displayed in log2. *Nos1ap* expression is significantly (P<0·001) increased ∼294 fold (∼8·2-fold in log2) in the hippocampi of NOS1AP (N=10 hippocampi from 5 mice), but not NOS1AP_396-503_ (N=8 hippocampi from 5 mice) injected mice (Kruskal-Wallis test: χ^2^(2)=18·621, P<0·001, ε^2^=0·69). (f, g) Co-immunoprecipitation (IP) using nNOS as a bait (for input samples see Fig. S2). (f) Example immunoblots for nNOS, NOS1AP, and PSD-95 on the nNOS co-IP samples. (g) Quantification of co-IP data showing reduced interaction of endogenous NOS1AP with nNOS (One-Way ANOVA: F_2,6_=33·09, P<0·001, η^2^=0·917) and increased interaction of PSD-95 with nNOS (One-Way ANOVA: F_2,6_=18·887, P=0·003, η^2^=0·863), for both NOS1AP (N=3) and NOS1AP_396-503_ (N=3) compared to mCherry (N=3). Abbreviations: hSyn = human *Synapsin 1* gene promoter, ITR = inverted terminal repeat, pA = human growth hormone polyadenylation signal, WPRE = woodchuck hepatitis virus posttranscriptional regulatory element. Box plots in (e) show median and interquartile range (IQR), and whiskers reflect the data range within 1.5 x IQR below or above the 1^st^ or 3^rd^ quartile respectively. Bar diagrams in (g) show arithmetic mean ± standard error of the mean. Individual data points are marked by grey circles, and outliers are marked in red. Asterisks indicate significant differences of uncorrected post-hoc tests: **P≤0·01, ***P≤0·001.

For the experiments described in this study, we stereotaxically delivered rAAVs encoding murine NOS1AP (NM_001109985, corresponding to the human full-length isoform NM_014697) [14] or the carboxyterminus of murine NOS1AP (i.e. NOS1AP_396-503_), required for nNOS interaction [21], both tagged with 3xFLAG and mCherry, to the dorsal hippocampus of wild-type C57BL/6JRj mice. An rAAV expressing mCherry and 3xFLAG was used as a control (Fig. 1a,b).

Analysis of mCherry fluorescence (Fig. 1c) confirmed high expression levels in all subregions of the dorsal hippocampus (Fig. S1). Congruent with previous findings [14] the NOS1AP rAAV had the lowest expression of the viruses. Ectopic expression was limited to the deep cortical layers above the injection site, comparable to previous observations [49]. Immunoblot analysis indicated that expression of the virally encoded proteins was substantially higher than endogenous NOS1AP levels (Fig. 1d), though these blots were not readily quantifiable due to signal saturation (note that with shortened exposure time, endogenous NOS1AP was not visible anymore [i.e. not quantifiable] and could not be used for reference). Expression analysis using qPCR (Fig. 1e, Table 1) showed ∼294-fold increase of *Nos1ap* mRNA in NOS1AP rAAV injected mice (Dunn's test, P<0·001). As the primers targeted the 5’- region of *Nos1ap* (corresponding to the aminoterminus), no expression changes were detected in NOS1AP_396-503_ mice (Dunn's test, P=0·5).

**Table 1:** Gene expression levels of *Nos1ap* and associated genes (see Fig. S3 for graphical representation)

Gene	mCherry	NOS1AP	NOS1AP_396-503_	Statistics^1^
*Cpe*	1·0e -10 ± 0·545	0·021 ± 0·33	0·772 ± 0·235	χ^2^(2)=2·472, P=0·29, ε^2^=0·092
*Dlg1*	-5·6e -18 ± 0·183	-0·412 ± 0·196	0·218 ± 0·154	χ^2^(2)=4·449, P=0·108, ε^2^=0·165
*Dlg3*	-3·1e -18 ± 0·187	0·051 ± 0·187	0·048 ± 0·311	χ^2^(2)=0·331, P=0·847, ε^2^=0·012
*Dlg4*	-1·9e -17 ± 0·214	0·23 ± 0·25	0·445 ± 0·264	χ^2^(2)=1·203, P=0·548, ε^2^=0·045
*Gria1*	1·0e -10 ± 0·261	0·051 ± 0·247	0·695 ± 0·289	χ^2^(2)=2·823, P=0·244, ε^2^=0·105
*Gria2*	4·163e -18 ± 0·251	-0·01 ± 0·157	0·506 ± 0·228	χ^2^(2)=3·03, P=0·22, ε^2^=0·112
*Grin2a*	-5·573e -18 ± 0·259	-0·183 ± 0·233	0·474 ± 0·196	χ^2^(2)=2·796, P=0·247, ε^2^=0·104
*Grin2b*	0·0 ± 0·491	0·739 ± 0·177	1·087 ± 0·211	χ^2^(2)=3·844, P=0·146, ε^2^=0·142
*Gucy1a1*	1·0e -10 ± 0·393	0·317 ± 0·144	0·63 ± 0·205	χ^2^(2)=1·822, P=0·402, ε^2^=0·067
*Gucy1a2*	-2·0e -10 ± 0·17	-0·463 ± 0·341	-0·204 ± 0·146	χ^2^(2)=1·81, P=0·404, ε^2^=0·067
*Gucy1b1*	-1·0e -10 ± 0·469	0·425 ± 0·179	0·947 ± 0·128	χ^2^(2)=5·617, P=0·06, ε^2^=0·208
*Map2k3*	1·0e -10 ± 0·39	0·56 ± 0·444	1·54 ± 0·241	χ^2^(2)=9·597, P=0·008, ε^2^=0·355
*Mapk14*	-2·2e -17 ± 0·501	0·311 ± 0·15	0·886 ± 0·182	χ^2^(2)=4·185, P=0·123, ε^2^=0·155
*Nos1*	-3·0e -10 ± 0·407	-0·368 ± 0·563	0·477 ± 0·358	χ^2^(2)=1·893, P=0·388, ε^2^=0·07
***Nos1ap***	**-1·0e -10 ± 0·191**	**8·199 ± 0·273**	**-0·168 ± 0·117**	**χ**^**2**^**(2)=18·621, P<0·001, ε**^**2**^**=0·69**
*Rasd1*	2·0e -10 ± 0·305	-0·253 ± 0·381	-0·645 ± 0·225	χ^2^(2)=1·975, P=0·373, ε^2^=0·073
*Scrib*	1·0e -10 ± 0·627	0·884 ± 0·213	1·574 ± 0·189	χ^2^(2)=7·967, P=0·019, ε^2^=0·295
*Syn1*	1·0e -10 ± 0·441	1·02 ± 0·295	1·044 ± 0·532	χ^2^(2)=5·749, P=0·056, ε^2^=0·213

1 Nominal (i.e. uncorrected) P-values from the Kruskal-Wallis test are shown. Targets below the Bonferroni-corrected significance threshold (P_Bonf_=0·00278) are indicated in bold.

We previously showed that virally-overexpressed NOS1AP and a short isoform of NOS1AP, similar to NOS1AP_396-503_, interact with endogenous nNOS [14]. Given their overabundance, endogenous nNOS appeared to be predominantly bound by these virally-expressed proteins, strongly reducing the interaction with endogenous NOS1AP, as shown by co-IP (Fig. 1f,g; LSD test, P<0·001 for NOS1AP and NOS1AP_396-503_). Importantly, levels of endogenous NOS1AP and nNOS were not significantly affected by viral overexpression, as suggested by semi-quantitative immunoblot analysis (Fig. S2a,b).

In keeping with recent findings suggesting that NOS1AP at least transiently mediates the function of the nNOS/PSD-95 NMDA receptor complex (discussed in [34]), using co-IP, we found that viral overexpression of NOS1AP and NOS1AP_396-503_ strongly increased the nNOS/PSD-95 interaction (Fig. 1f,g; LSD test, P=0·002 for both) while overall PSD-95 levels were not significantly affected (Fig. S2c).

In addition, we wanted to investigate if NOS1AP or NOS1AP_396-503_ overexpression affects the expression of NOS1AP-associated genes. We found that the expression of *Map2k3* and *Scrib* was nominally affected (Kruskal-Wallis test, P=0·008 and P=0·019 respectively), but not after Bonferroni correction (P_Bonf_=0·00278). Analysis with removed outliers resulted in *Syn1* expression passing the nominal significance threshold (Kruskal-Wallis test, P=0·015) and *Map2k3* expression passing the Bonferroni corrected significance threshold (Kruskal-Wallis test, P=0·002) with post-hoc comparison suggesting a significant increase of *Map2k3* in NOS1AP_396-503_ compared to mCherry (Dunn's test, P<0·001) and NOS1AP (Dunn's test, P=0·012) mice. The expression of all other tested genes was not significantly affected (Kruskal-Wallis test, P>0·05; Table 1 and Fig. S3), suggesting that the treatment only had a limited effect on the regulation of these genes.

### 3.2 Hippocampal overexpression of NOS1AP changes dendritic spine morphology

**Fig. 2:**
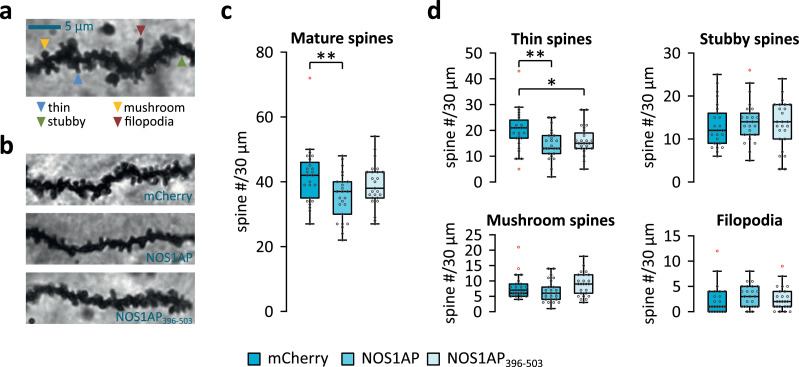
Overexpression of NOS1AP reduces the spine number of CA1 neurons. Dendrites of Golgi-impregnated brains from mice injected with mCherry, NOS1AP, or NOS1AP_396-503_ (for all groups N=25 dendrites from 5 mice) were quantified for dendritic spines, including classification into different spine types. (a) Example image highlighting the different spine types analysed herein. (b) Example images of Golgi impregnated dendrites from mice injected with the mCherry, NOS1AP, or NOS1AP_396-503_ expressing virus. (c) The total number of mature spines was significantly reduced in neurons overexpressing NOS1AP, but not in those expressing NOS1AP_396-503_ (Kruskal-Wallis test: χ^2^(2)=7·164, P=0·028, ε^2^=0·097). (d) Analysis for spine types showed a significant reduction of thin spines in mice expressing NOS1AP or NOS1AP_396-503_ (Kruskal-Wallis test: χ^2^(2)=9·187, P=0·01, ε^2^=0·124), but not stubby (Kruskal-Wallis test: χ^2^(2)=0·692, P=0·708, ε^2^=0·009) or mushroom (Kruskal-Wallis test: χ^2^(2)=5·093, P=0·078, ε^2^=0·069) spines. No changes in filopodia-like protrusions were detected (Kruskal-Wallis test: χ^2^(2)=2·712, P=0·258). Box plots in (c) and (d) show median and interquartile range (IQR), and whiskers reflect the data range within 1.5 x IQR below or above the 1^st^ or 3^rd^ quartile respectively. Individual data points are marked by grey circles, and outliers are marked in red. Asterisks indicate significant differences in uncorrected post-hoc Dunn's tests: *P≤0·05, **P≤0·01.

We previously showed that overexpression of murine NOS1AP in cultured primary neurons resulted in an increase in filopodia and a reduction in dendritic spines, particularly thin and mushroom spines [14]. Thus, to investigate whether overexpression of NOS1AP also affects dendritic spine development in vivo, we quantified dendritic spines in Golgi impregnated dorsal hippocampal CA1 neurons of mice with dorsal hippocampus injections (Fig. 2a-d). We focused on the CA1 as the primary output region of the hippocampus, where changes in activity in the other subregions are expected to propagate to [60]. In agreement with our in vitro study [14], we found that the total number of mature spines (i.e. excluding filopodia) was significantly reduced in brains overexpressing NOS1AP (Dunn's test, P=0·004) and a strong trend for reduction in those expressing NOS1AP_396-503_ (Dunn's test, P=0·052) when compared to control brains (Fig. 2c).

Functional properties of dendritic spines correlate strongly with their morphology [19]. Thus, we further analysed different spine classes according to their morphology (i.e. thin, stubby, mushroom) (Fig. 2d). We found a significant reduction in thin spines in brains overexpressing NOS1AP (Dunn's test, P=0·002) or NOS1AP_396-503_ (Dunn's test, P=0·019), but no changes in the number of stubby (Kruskal-Wallis test, P=0·708) or mushroom (Kruskal-Wallis test, P=0·078) spines, or filopodia (Kruskal-Wallis test, P=0·258). In all cases, analysis with removed outliers yielded comparable results (data not shown).

### 3.3 Overexpression of NOS1AP in dorsal hippocampus disrupts selective behaviours related to mental disorders

Given the substantial effects of viral NOS1AP and NOS1AP_396-503_ expression on nNOS/PSD-95 interaction and dendritic spines, we further investigated their effect on behaviour.

**Fig. 3:**
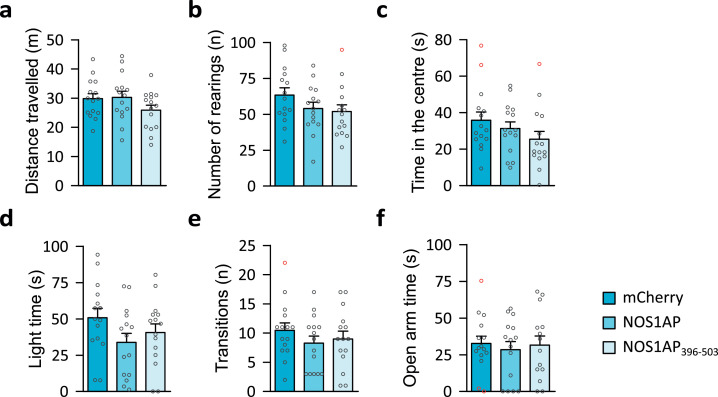
Basic behavioural phenotypes were not affected by overexpression of NOS1AP or NOS1AP_396-503_. (a) Distance travelled (One-Way ANOVA: F_2,42_=1·725, P=0·191, η^2^=0·076) and (b) number of rearings (One-Way ANOVA: F_2,42_=1·665, P=0·202, η^2^=0·073) in the open field were comparable in all groups (N=15/group). (c) The time spent in the centre of the open field was also unaffected (Kruskal-Wallis test: χ^2^(2)=4·005, P=0·135, ε^2^=0·091). (d) The time spent in the light compartment (F_2,42_=1·891, P=0·164, η^2^=0·083) and (e) the number of transitions (One-Way ANOVA: F_2,42_=0·793, P=0·459, η^2^=0·036) in the light-dark-box were comparable across groups (N=15/group). (f) Mice from all groups (mCherry: N=15, NOS1AP: N=15, NOS1AP_396-503_: N=14) spent a comparable amount of time in the open segments of an elevated zero maze (One-Way ANOVA: F_2,41_=0·154, P=0·858, η^2^=0·007). All data are shown in bar diagrams which reflect the arithmetic mean ± standard error of the mean. Individual data points are marked by grey circles, and outliers are marked in red.

Overexpression of NOS1AP or NOS1AP_396-503_ did not affect basic behavioural phenotypes (Fig. 3). Specifically, horizontal (distance travelled: Fig. 3a, One-Way ANOVA, P=0·191) and vertical (number of rearings: Fig. 3b, One-Way ANOVA, P=0·202) activity in the open field were comparable across all groups. Analysis with removed outliers revealed comparable results. Previous studies in mice of the ICR strain have suggested that NOS1AP overexpression in the dentate gyrus, targeting a region more posterior to the one used here, had anxiogenic effects [13]. However, in our study, anxiety-related behaviours in the open field (time in the centre: Fig. 3c, One-Way ANOVA, P=0·135), the light dark-box (light time: Fig. 3d, One-Way ANOVA, P=0·164; number of transitions: Fig. 3e, One-Way ANOVA, P=0·459), and the elevated zero maze (open arm time: Fig. 3f, One-Way ANOVA, P=0·858) were not affected by NOS1AP/NOS1AP_396-503_ overexpression. Removing outliers did not change the outcome of any of these measures (data not shown).

**Fig. 4:**
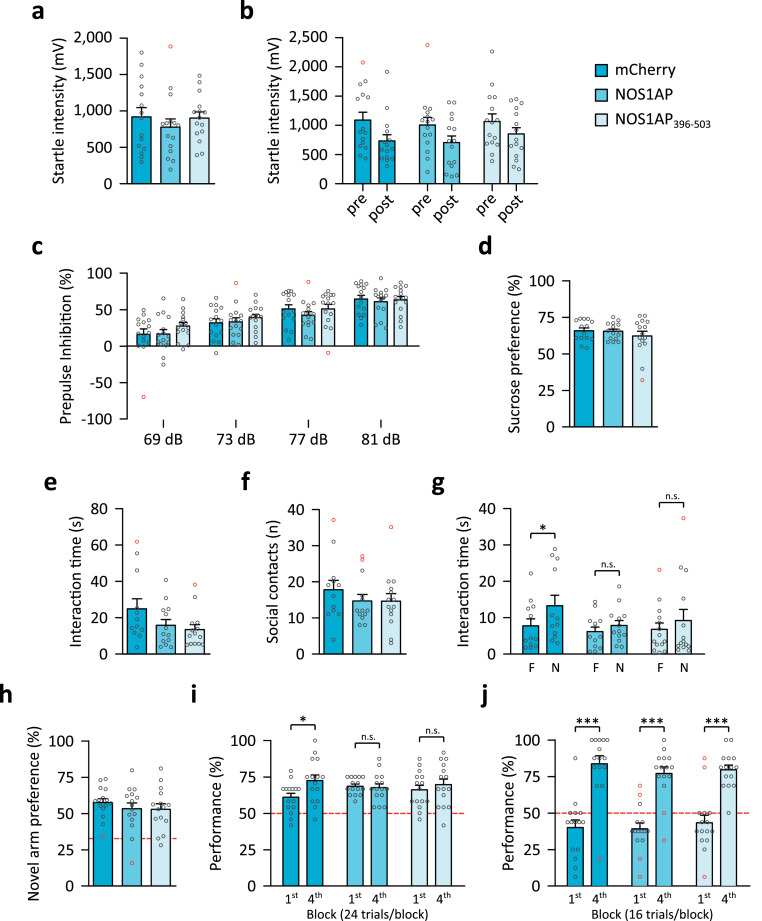
Overexpression of NOS1AP in mouse hippocampus changes some behavioural phenotypes related to mental disorders. (a) The startle response was not affected (One-Way ANOVA: F_2,42_=0·498, P=0·611, η^2^=0·023) in any of the groups (N=15/group). (b) Startle habituation was intact in all groups (N=15/group) with reduced startle intensity at the end of the session (post) compared to the beginning (pre) (repeated measures ANOVA: F_1,42_=19·019, P<0·001, η^2^=0·092) and not affected by the treatment (F_2,42_=0·241, P=0·787, η^2^=0·008) or the interaction (F_2,42_=0·385, P=0·683, η^2^=0·004). (c) Prepulse inhibition (PPI) increased with prepulse intensity (Friedman test: mCherry, χ^2^(3)=40·44, P<0.001, ε^2^=0·919; NOS1AP, χ^2^(3)=30·6, P<0.001, ε^2^=0·695; NOS1AP_396-503_, χ^2^(3)=34·36, P<0.001, ε^2^=0·781), but was not affected by the treatment (Kruskal-Wallis test: 69 dB, χ^2^(2)=2·081, P=0·353, ε^2^=0·047; 73 dB, χ^2^(2)=1·355, P=0·508, ε^2^=0·031; 77 dB, χ^2^(2)=2·451, P=0·294, ε^2^=0·056; 81 dB, χ^2^(2)=0·392, P=0·822, ε^2^=0·009) suggesting intact sensorimotor gating (N=15/group). (d) Anhedonia, as measured by sucrose preference (mCherry: N=14, NOS1AP: N=15, NOS1AP_396-503_: N=15), was not influenced by our treatment (Kruskal-Wallis test: χ^2^(2)=0·336, P=0·845, ε^2^=0·008). (e, f) None of the tested groups (mCherry: N=12, NOS1AP: N=13, NOS1AP_396-503_: N=14) showed changes in (e) social interaction time (Kruskal-Wallis test: χ^2^(2)=3·695, P=0·158, ε^2^=0·097) or (f) the number of social contacts (Kruskal-Wallis test: χ^2^(2)=1·442, P=0·486, ε^2^=0·038) when exposed to a novel juvenile conspecific. (g) Recognition of a novel (N) over the familiar (F) conspecific was tested 30 min after the social interaction paradigm. No significant between group differences were detected for both the interaction with the familiar (Kruskal-Wallis test: χ^2^(2)=0·338, P=0·845, ε^2^=0·009) and the novel conspecific (Kruskal-Wallis test: χ^2^(2)=4·222, P=0·121, ε^2^=0·111). Within group analysis showed that control mice expressing mCherry (N=13) showed intact recognition of the novel compared to the familiar juvenile conspecific (Wilcoxon signed-rank test: W=70, P=0·012, r_B_=0·795). Mice overexpressing NOS1AP (N=14; Wilcoxon signed-rank test: W=65, P=0·184, r_B_=-0·429) or NOS1AP_396-503_ (N=15; Wilcoxon signed-rank test: W=56, P=0·485, r_B_=-0·231) displayed comparable investigation time of the familiar and novel conspecifics, suggesting deficient social recognition. (h) Novel arm preference was comparable in all groups (N=15/group) suggesting overall intact SWM (One-Way ANOVA: F_2,42_=0·517, P=0·6, η^2^=0·024). (i) In the rewarded alternation paradigm on the T-maze control mice (i.e. mCherry, N=15) significantly improved performance throughout training (4^th^ vs 1^st^ block, Wilcoxon signed-rank test: W=74, P=0·049, r_B_=0·626) suggesting an increase in SWM capacity. Mice overexpressing NOS1AP (N=15; Wilcoxon signed-rank test: W=31, P=0·893, r_B_=-0·061) or NOS1AP_396-503_ (N=15; Wilcoxon signed-rank test: W=59, P=0·363, r_B_=0·297) showed comparable performance throughout training, suggesting a limited increase in SWM capacity (Kruskal-Wallis test: 1^st^ block, χ^2^(2)=3·923, P=0·141, ε^2^=0·089; 4^th^ block, χ^2^(2)=1·108, P=0·575, ε^2^=0·025). (j) Spatial reference memory, measured in the Y-maze, was intact in mice from all groups (N=15/group), as performance significantly improved throughout training (Wilcoxon signed-rank test 1^st^ vs 4^th^ block: mCherry, W=120, P<0·001, r_B_=1·0; NOS1AP, W=118·5, P<0·001, r_B_=0·975; NOS1AP_396-503_, W=105, P=0·001, r_B_=1·0) and no differences between groups were found (Kruskal-Wallis test: 1^st^ block, χ^2^(2)=0·371, P=0·831, ε^2^=0·008; 4^th^ block, χ^2^(2)=3·023, P=0·221, ε^2^=0·069). (h-j) Chance level performance is indicated by the red dashed lines. All data are shown in bar diagrams which reflect the arithmetic mean ± standard error of the mean. Individual data points are marked by grey circles, and outliers are marked in red. Asterisks indicate significant differences with uncorrected post-hoc comparison: *P≤0·05, ***P≤0·001.

Sensorimotor gating, operationalized by PPI of the ASR is impaired in schizophrenia and other mental disorders (reviewed in [61]) and *NOS1AP* variants affecting PPI and startle have been identified [8]. Startle reactivity (Fig. 4a) was comparable in all groups (One-Way ANOVA, P=0·611) and startle habituation (Fig. 4b) was intact in all groups (One-Way ANOVA, P<0·001), with no effect of treatment (One-Way ANOVA, P=0·787) or the interaction (One-Way ANOVA, P=0·683). PPI significantly increased with prepulse intensity (Fig. 4c, Friedman test, P≤0·001 for all groups), but was unaffected by the treatment (Kruskal-Wallis test, P>0·29 for all prepulse intensities) indicating that hippocampal NOS1AP/NOS1AP_396-503_ overexpression does not influence sensorimotor gating. Analysis without outliers revealed similar findings for these measures (data not shown).

NOS1AP was previously linked to depression and depression phenotypes, but not specifically within the hippocampus [6,10]. Therefore, we tested our mice for anhedonia, a common negative symptom caused by impairments in reward-related pathways including the hippocampus [62]. However, no differences in anhedonia, measured by sucrose preference, were observed between groups (Kruskal-Wallis test, P=0·845; Fig. 4d). Sucrose preference was also not affected when outliers were removed (data not shown).

Deficits in sociability and social cognition are commonly found in autism spectrum disorders [63], schizophrenia [64] and other mental disorders including depression [65]. When exposed to a juvenile conspecific, social interaction time (Fig. 4e; Kruskal-Wallis test, P=0·158) and the number of social contacts (Fig. 4f; Kruskal-Wallis test, P=0·486) were not affected in any of the groups. Analysis with outliers excluded revealed comparable results (data not shown). Thirty min later mice were tested for social memory by exposure to the same (‘familiar’) and an unfamiliar (‘novel’) juvenile conspecific (Fig. 4g). No between group differences for interaction with the familiar (Kruskal-Wallis test, P=0·845) or novel (Kruskal-Wallis test, P=0·121) conspecific were detected (though analysis without outliers suggested a significant [Kruskal-Wallis test, P=0·046] effect for the novel conspecific with significant reduction in the NOS1AP_396-503_ group [Dunn's test, P=0·007] and a trend for significance in the NOS1AP group [Dunn's test, P=0·087]). However, we observed significantly longer interaction with the novel mouse in controls (Wilcoxon signed-rank test, P=0·012), while NOS1AP and NOS1AP_396-503_ overexpressing mice showed no preference for the novel mouse (Wilcoxon signed-rank test, P=0·184 and P=0·485 respectively). These findings show that viral overexpression of NOS1AP and NOS1AP_396-503_ in hippocampus leads to deficits in social memory.

Deficits in working memory have been proposed as a transdiagnostic endophenotype for mental disorders [66]. Performance in the novel arm paradigm, a test requiring limited SWM capacity [49] was not affected (Fig. 4h, P=0·6), suggesting that NOS1AP/NOS1AP_396-503_ overexpression does not influence basic SWM. Confirming this observation, mice from all groups performed well above chance level with comparable performance across groups (Kruskal-Wallis test, 1^st^ block: P=0·141; 4^th^ block: P=0·575), in the rewarded alternation paradigm on the T-maze (Fig. 4i). Control mice improved SWM performance (Wilcoxon signed-rank test, P=0·049) across training. In contrast, neither NOS1AP (Wilcoxon signed-rank test, P=0·893) nor NOS1AP_396-503_ (Wilcoxon signed-rank test, P=0·363) expressing mice showed a significant increase in SWM performance indicating limited SWM capacity. This deficit cannot be attributed to an overall spatial memory deficit, as spatial reference memory was intact in all groups (Fig. 4j, Wilcoxon signed-rank test, P≤0·001 for all groups) and no differences between groups were detected (Kruskal-Wallis test, 1^st^ block: P=0·831; 4^th^ block: P=0·221). Removing outliers did not change the outcome of these analyses (data not shown).

## 3.4 Discussion

The adaptor protein NOS1AP, which interacts with the nNOS/PSD-95/NMDA receptor complex and links nNOS to downstream pathways, thus mediating their activation [34], has been linked to multiple mental disorders including depression, schizophrenia, PTSD, and related (endo)phenotypes [1,2,[6], [7], [8], [9], [10], [11], [12],^67^]. Here we created a targeted overexpression mouse model and revealed that hippocampal NOS1AP overexpression led to changes in dendritic spine morphology and selective behavioural abnormalities. Increased hippocampal NOS1AP might thus give rise to a specifically altered behaviour that contributes to mental disorders in a transdiagnostic manner, pinpointing the network-specific molecular underpinnings of these cross-disorder symptoms.

Strong support for a functional role of NOS1AP to various mental disorder (endo)phenotypes was provided by the COGS family study [7], [8], [9]. Translating our data to the human situation, the behavioural changes observed upon NOS1AP overexpression partially overlap with endophenotypes implicated by the COGS study (e.g. social interaction/memory, SWM), while other domains remained unaffected (e.g. sensorimotor gating, anhedonia, spatial reference memory). As the human COGS study examined genotypes, but not brain regions, one may speculate that the latter domains might be a consequence of non-hippocampal NOS1AP expression changes.

To date, despite preclinical evidence revealing that genetic or pharmacological disruption of nNOS/NOS1AP interactions targeting an intermediate region of the dentate gyrus (and parts of CA3) has anxiolytic effects [13,[68], [69], [70]], there is no study linking NOS1AP to anxiety in humans. Consistent with this lack of clinical association, in the present study overexpression of NOS1AP throughout the dorsal hippocampus did not affect anxiety-related behaviours. Thus, these differences may be a result of region-specific contributions and the differential role of the hippocampus along the dorsoventral axis [71], [72], [73]. Likewise, while NOS1AP has been linked to depression (though not yet in the hippocampus, but the prefrontal cortex) [6], we found no effect on depression-related behaviours. These findings further suggests that NOS1AP is linked to various behaviours depending on the neural circuit it is involved in.

Critically, the selective deficits observed in our study are precisely in line with our expectations given (i) the broad NOS1AP expression pattern, (ii) the reported NOS1AP overexpression in other brain regions described in patients with schizophrenia [4,5] and depression [6], (iii) the differential contribution of the hippocampus along its longitudinal axis (e.g. reviewed in [44]) (iv) the high variability of symptoms in patients, and (v) the vast number of other molecules/pathways associated with mental disorders [39]. In fact, using this selective type of manipulation we can more clearly discern the specific effect of elevated NOS1AP encompassing all subregions of the dorsal hippocampus (i.e. CA1, CA3, and dentate gyrus) than with other rather unphysiological manipulations such as global gene overexpression or knockout. Subtle variations that specifically manipulate gene expression in selected circuits may better reflect the human situation [74].

Dendritic spines increase in size and become more stable with persistent stimulation [75]. The relatively large and stable mushroom spines have been considered ‘memory spines’, while the more transient thin spines have been regarded as ‘learning spines’ [76]. Thus, our findings suggest that overexpression of NOS1AP and NOS1AP_396-503_ results in a loss of spines required for plasticity (i.e. thin spines) and thus learning, while memory-related spines (i.e. mushroom spines) remain unaffected. These findings are in line with our findings indicating compromised social memory and SWM capacity, but persistent spatial reference memory, as well as with in vitro data showing reduced dendritic growth and aberrant morphology of dendritic spines following overexpression of NOS1AP [14,15,17].

In addition to NOS1AP, we overexpressed its carboxyterminus (i.e. NOS1AP_396-503_), containing the nNOS interacting region [21]. While full-length NOS1AP can interact with downstream effectors (e.g. RasD1, MKK3) linking them to nNOS, NOS1AP_396-503_, similar to the short form of NOS1AP [4], lacks the phosphotyrosine-binding (PTB) domain and is therefore unable to interact with these proteins. Notably, most deficits in NOS1AP overexpressing mice were also observed in NOS1AP_396-503_ mice. The dramatic increase in nNOS/PSD-95 interaction observed in both groups is, most likely, the underlying reason for this. Importantly, PSD-95 has been suggested as a critical synaptic mediator of schizophrenia-related molecular consequences [77] and our previous study has shown that integrity of the NOS1AP/nNOS/PSD-95 complex is critical for the effect of NOS1AP on dendritic growth and spine plasticity [14]. Thus, downstream effectors such as RasD1or MKK3 might have a less direct role in the regulation of the (endo)phenotypes assessed here, as also supported by the lack of expression changes in NOS1AP interactions partners. However, it should be noted that, interactions through the PTB domain and other binding regions (e.g. that for CPE) have been shown to contribute to some of the effects mediated by NOS1AP [15,16].

As described above, a previous study by Zhu et al. [13] found that hippocampal overexpression of NOS1AP in the dentate gyrus had anxiogenic effects. Of note, in the same study, overexpression of the NOS1AP carboxyterminus (specifically the last 125 amino acids) resulted in anxiolysis. Thus, in contrast to our study overexpression of NOS1AP or its carboxyterminal tail had opposite effects (though on behaviours not affected in our study). This difference might in part be explained by the more limited region targeted in the study by Zhu et al. (i.e. dentate gyrus and parts of CA3 with limited viral spread due to the use of the larger lentivirus), as well as several factors that might differentially affect the binding dynamics of the overexpressed NOS1AP. These include lower expression levels, carboxyterminal localization of the fluorescent tag, and potential dimerization of GFP (also see discussion below).

We and others have previously emphasized the potential for targeting the nNOS/PSD-95 interaction for the pharmacological treatment of schizophrenia, depression, and other mental disorders [1,14,78,79]. The findings from the present study support this assertion and indicate a potential for directly targeting (i.e. disrupting) the interaction between NOS1AP with nNOS (or possibly other downstream effectors) using small molecules that are able to pass the blood brain barrier. Such treatments will likely not adhere to diagnostic boundaries such as ‘schizophrenia’ or ‘depression’, but target defined neuropsychological deficits (here, compromised working memory and social interaction) in conjunction with evidence for abnormalities in NOS1AP interactions, in the sense of precision medicine approaches.

In the present study, we have artificially increased NOS1AP selectively in dorsal hippocampus. This resulted in an overall increase in nNOS/NOS1AP interaction properly modelling the effect of pathophysiologically increased NOS1AP. However, under pathophysiological conditions NOS1AP expression is also likely to change in other brain regions and other genes, gene products, and molecular pathways will also be affected. For, example chronic mild stress in mice increased NOS1AP in hippocampus [13,69], but also in the prefrontal cortex [6], and decreased NOS1AP in the cingulate cortex [6]. Moreover, chronic mild stress has been shown to induce a broad range of molecular changes in different brain regions (e.g. [80], [81], [82]). Thus, we cannot conclusively confirm that the described effects following hippocampal overexpression of NOS1AP would also be observed as a consequence of pathophysiologically increased NOS1AP. Addressing this aspect will require further validation in relevant animal models, where NOS1AP is selectively downregulated. Of note, downregulation of NOS1AP in prefrontal cortex was shown to normalize depression-related behaviours in mice exposed to chronic mild stress [6]. Alternatively, as suggested above, NOS1AP protein interactions could be selectively inhibited. For example, inhibition of NOS1AP/nNOS interactions normalized anxiety related behaviours caused by chronic mild stress [13,70]. This latter study also showed that infusion of peptide or small molecule inhibitors recovered spine deficits observed following chronic mild stress [70], which fits well in the context of the present study, where NOS1AP overexpression caused spine deficits.

The above-mentioned studies investigating NOS1AP expression in mice after chronic mild stress showed ∼1.5-2.5-fold increased hippocampal NOS1AP protein levels [13,69] and a ∼1.7-fold increase in NOS1AP protein in the prefrontal cortex [6]. These results conform well with reports from human subjects showing that NOS1AP expression in the dorsolateral prefrontal cortex increases ∼1.5-fold [4] in patients with schizophrenia and ∼3-fold in patients with depression [6]. Of note, one study showed a ∼100-fold increase of NOS1AP expression in the dorsolateral prefrontal cortex of patients with schizophrenia [5]. In the present study we achieved a comparably high level of NOS1AP in the overexpression group (i.e. ∼296-fold increase in *Nos1ap* mRNA compared to the mCherry control). Although it was not possible to reliably quantify protein levels, our immunoblot analyses do indeed confirm high levels of overexpressed protein (though it is possible that the actual protein levels are somewhat lower than suggested by the level of mRNA, given the reduced stability of the viral mRNA due to the minimal stabilizing sequences included in the viral vectors [83,84]). Thus, the overexpression levels achieved in the present study are unlikely to fully reflect real (patho)physiological conditions. While this is to be expected when artificially modulating the expression of target genes (e.g. consider gene knockouts, where gene expression is reduced to 0%), it is conceivable that some of the effects described here might only be caused by these artificially high levels of NOS1AP and therefore might not occur as a consequence of pathophysiologically increased NOS1AP levels (for discussion also see [85]). Moreover, similar to what has been proposed for MAPK scaffolds [86,87] it is possible that high levels of NOS1AP may negatively impact adequate formation of a trimolecular complex consisting of nNOS, NOS1AP, and downstream effectors.

We have designed our viral vectors in a way to minimize a potential influence by the mCherry.3XFLAG tag. Specifically, we used a monomeric fluorophore to avoid oligomerization of the fluorescent tag [88,89] and placed the tag on the aminoterminal end to reduce interference with the nNOS binding region located at the carboxyterminus [21]. Moreover, we placed the 3XFLAG tag in between mCherry and NOS1AP/NOS1AP_396-503_ to serve as a linker of 26 amino acids length (22 amino acids for the 3XFLAG + 4 amino acids cloning artefacts). However, we cannot fully rule out a potential influence of the mCherry.3xFLAG tag on the function of the expressed NOS1AP/NOS1AP_396-503_. Although, we have previously shown that the overexpressed proteins interact with endogenous nNOS [14], it remains possible that the tag does influence the binding dynamics of the overexpressed NOS1AP/NOS1AP_396-503_ as we have not directly compared them with an untagged version of these proteins. Moreover, we cannot rule out that other protein interactions, e.g. those involving the aminoterminal PTB domain, might be influenced by the mCherry.3xFLAG tag.

Another potential limitation is that we only investigated the hippocampal CA1 subregion for changes in spine morphology. The CA1 subregion is the major output area of the hippocampus where most of the signals that are processed in the hippocampal trisynaptic circuit converge. Thus, changes in activity in dentate gyrus and CA3 are expected to project and propagate into the CA1 [60]. Only few studies investigating spine density in all hippocampal subregions exist. Of those, in several cases, manipulations that affected CA1 spine density also similarly affected spine density in the dentate gyrus and CA3 (see. e.g. [90,91]). However, in other cases a specific manipulation only affected spine density in some of the hippocampal subregions (see e.g. [92,93]). Thus, we cannot rule out the possibility that our manipulation differentially affected spine density in dentate gyrus and/or CA3.

Taken together, we demonstrate a potential importance of hippocampal NOS1AP in the pathophysiology of different mental disorders. Through virus-mediated NOS1AP overexpression in the dorsal hippocampus of mice we recapitulated alterations in dendritic spine morphology also observed e.g. in patients with schizophrenia and mood disorders. Moreover, we reveal a role of NOS1AP overexpression in select (endo)phenotypes of psychiatric conditions including social memory and SWM capacity, without impacting on other aspects, such as PPI or anhedonia. Thus, this study enabled us to distinctly identify the contribution of hippocampal NOS1AP to phenotypes related to mental disorders. NOS1AP may thus provide an attractive target for disease stratification and targeting of NOS1AP protein interactions may be a potential target for novel pharmacological interventions at least in a subpopulation of patients.

## Contributors

FF and EC cloned, purified, and validated the viral constructs and performed stereotaxic surgeries. FF, AOL, and DAS designed, and FF and EC performed mouse behavioural experiments and analysed behavioural data. FF, LG, and MK performed and analysed qPCR experiments. LLL, SB, and MJC performed Western blot and co-IP analyses and LLL and MJC analysed data. DES, NS, VF, and NF performed Golgi staining and quantified dendritic spines on Golgi-stained brain slices. EC, JM, SB, and AK sliced brains and microscopically analysed brain slices. EC, XC, JvE, and FF analysed data. FF and AR conceived and supervised all experiments and verified the underlying data. FF and AR wrote the first draft of the manuscript. All authors contributed to and proofread the manuscript and approved the final version.

## Declaration of competing interest

Andreas Reif received speaker's honoraria from Janssen, Medice, Shire/Takeda, Servier and neuraxpharm, is on the Advisory boards for Janssen, Medice, Shire/Takeda and SAGE and received grant support from Medice. None of these relationships are directly related to the study reported herein. All other authors report no financial relationships with commercial interests.

## Appendix Supplementary materials

Image, application 1

Image, application 2

Image, application 3

Image, application 4

Image, application 5

Image, application 6

Image, application 7
