# Supplementary material for: Hippocampal overexpression of NOS1AP promotes endophenotypes related to mental disorders
Source: eBioMedicine. 2021 Aug 27;71:103565. doi: 10.1016/j.ebiom.2021.103565 (PMC8403735; doi:10.1016/j.ebiom.2021.103565)

**Original blots for Fig.1 d & f and Fig. S2**

The entire film pieces used for exposing the blots are shown.

Input samples are the brain lysates used in the co-immunoprecipitation (co-IP) experiments. When NOS1AP antibody was used for blotting the input samples, the exogenous expressed mCherry-NOS1AP fusions (NOS1AP and NOS1AP_396-503_) were strongly detected. However, such strong signals interfered with the detection of endogenous NOS1AP due to uneven depletion of substrate and fogging of the films. Thus, the blots were cut to remove the strong bands and re-exposed to ECL reagents to detect the endogenous NOS1AP.

For co-IP samples, blots were cut above the 95 kDa marker, the upper part was used for nNOS detection and the lower part for NOS1AP. For this reason the overexpressed mCherry-NOS1AP, which runs at about 100kD was, in most cases, not or barely detected on the cut blots. The lower parts of the blots were also cut at about the 55 kDa marker to remove both the overexpressed mCherrry.3XFLAG.NOS1AP_396-503_ band and the heavy chain of the co-IP antibody.

The order of the samples on all blots are uninjected, mCherry, NOS1AP and NOS1AP_396-503_.

**Set 1 Input samples:**

nNOS


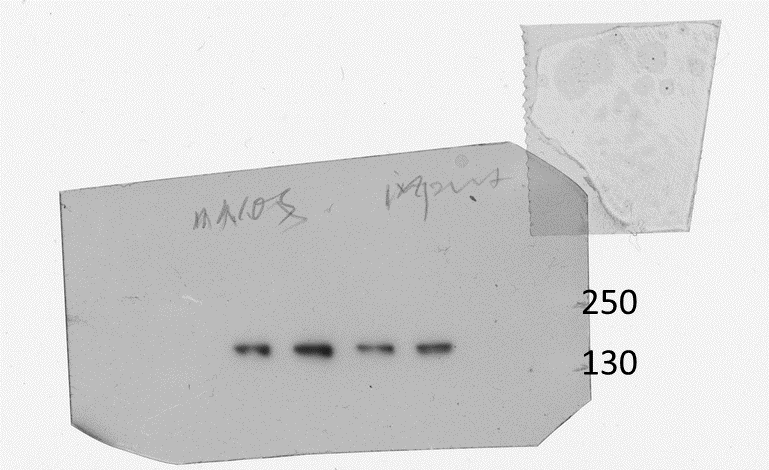


PSD95


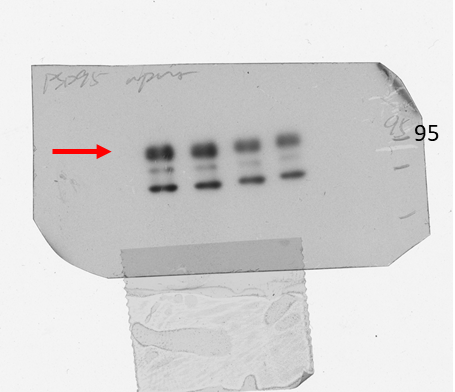


NOS1AP


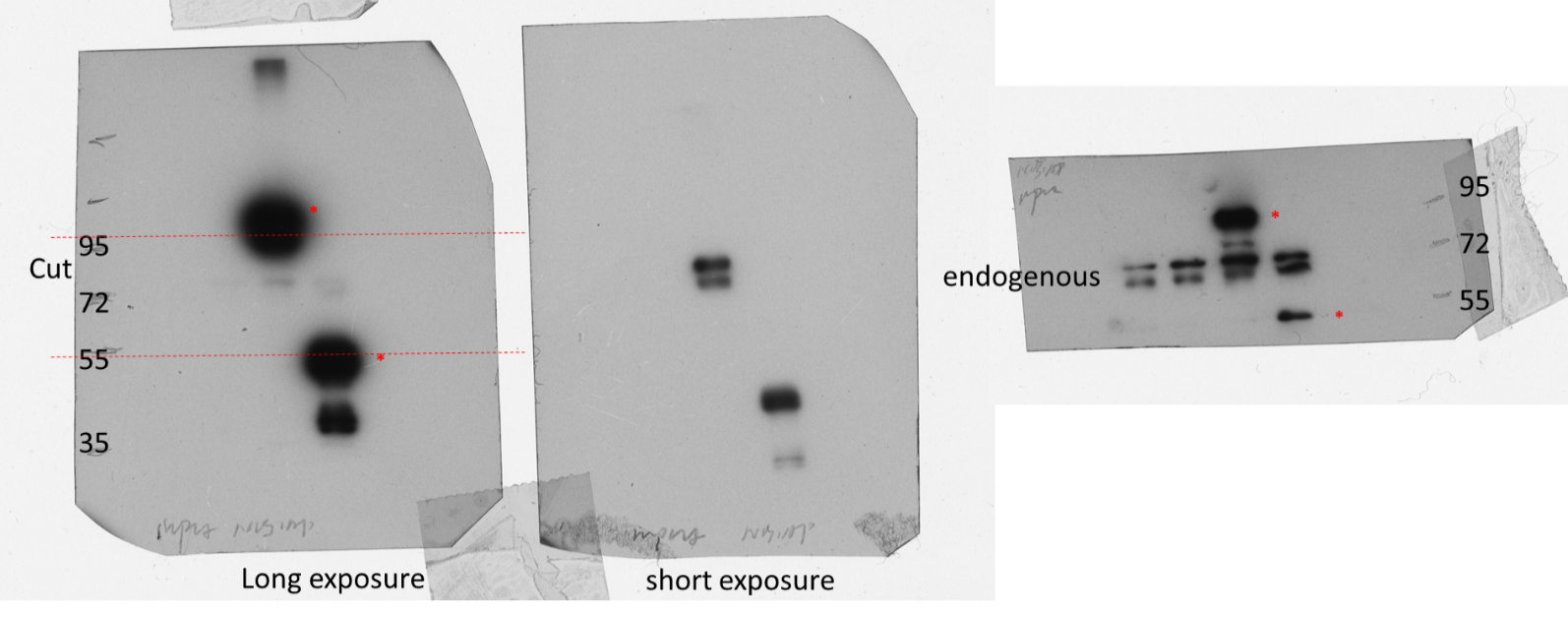


**Set1 Co-IP samples:**

nNOS


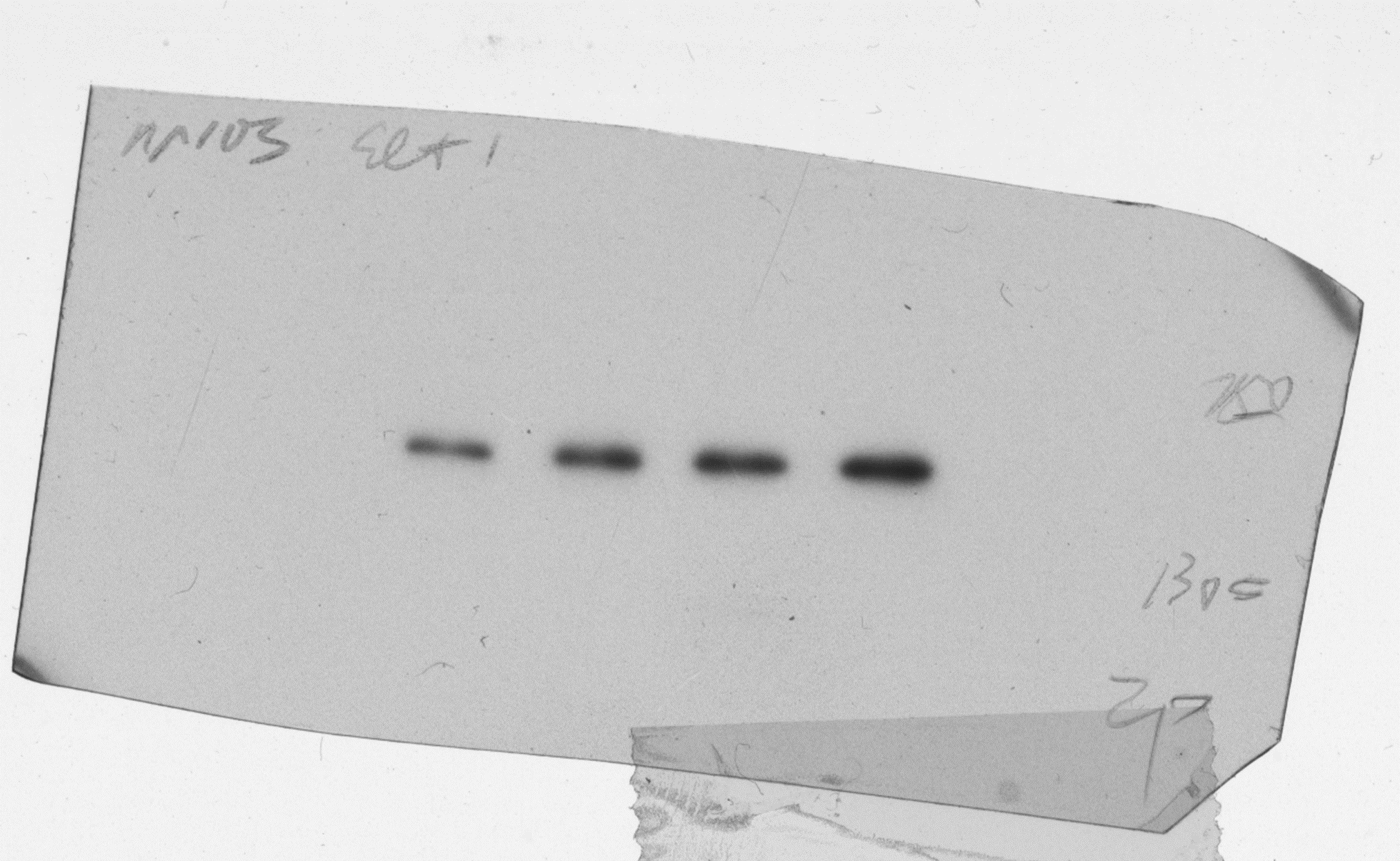


PSD95


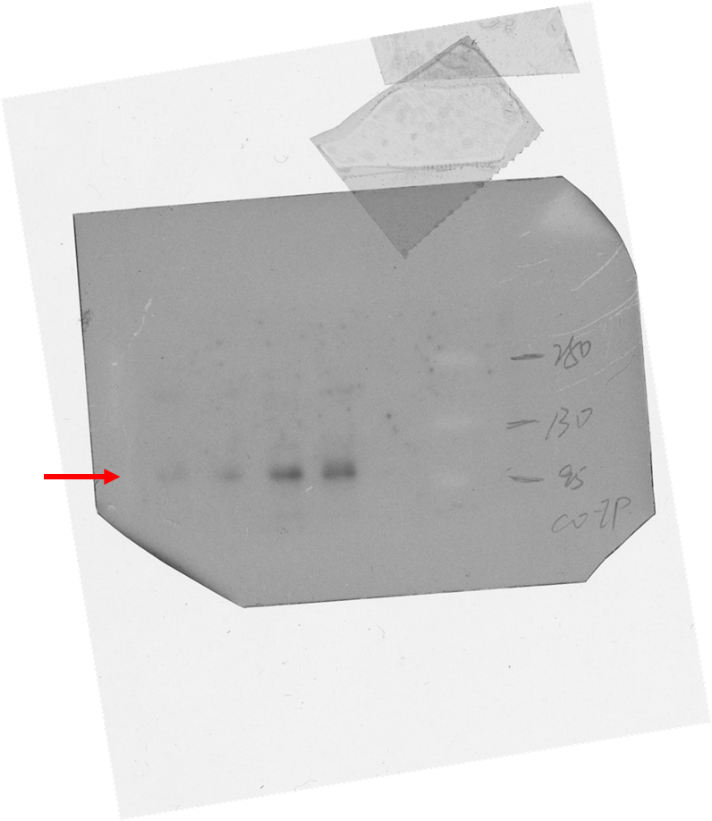


NOS1AP
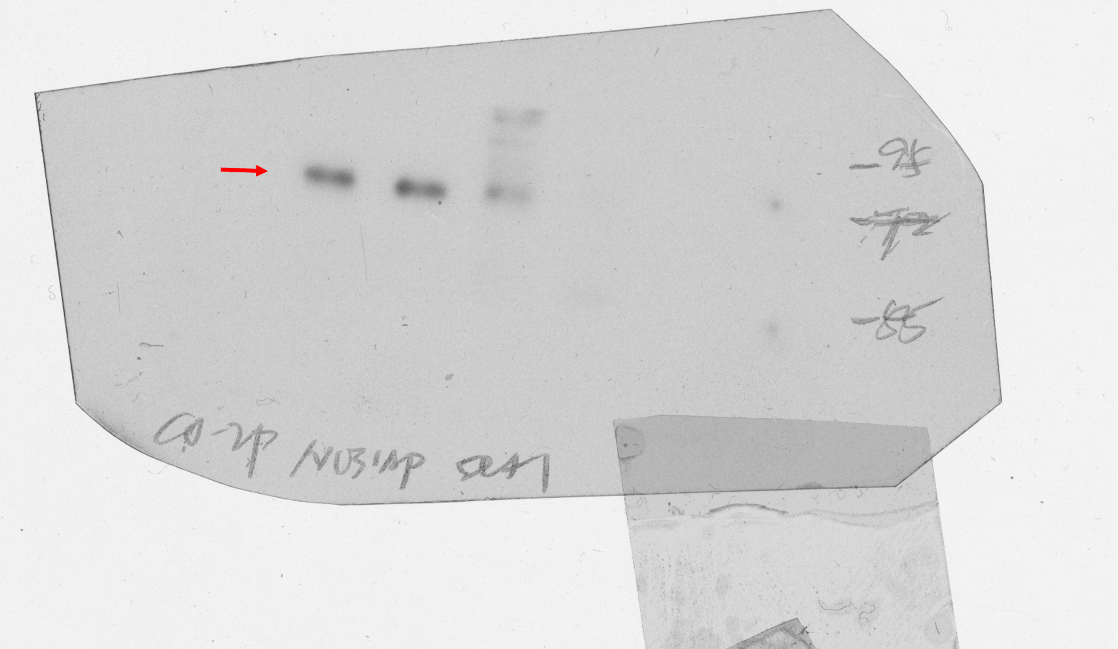


**Set 2 Input samples:**

nNOS


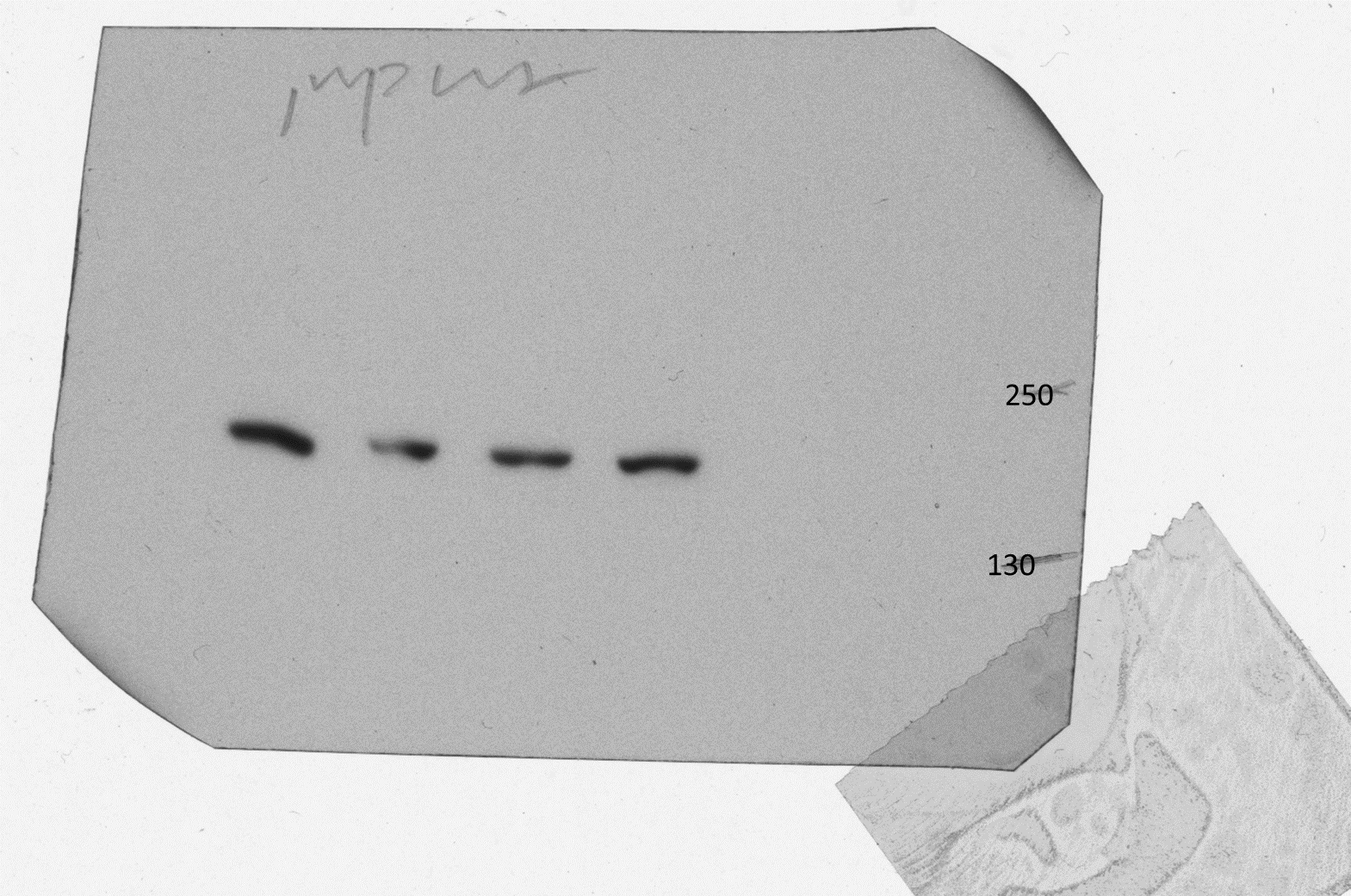


PSD95


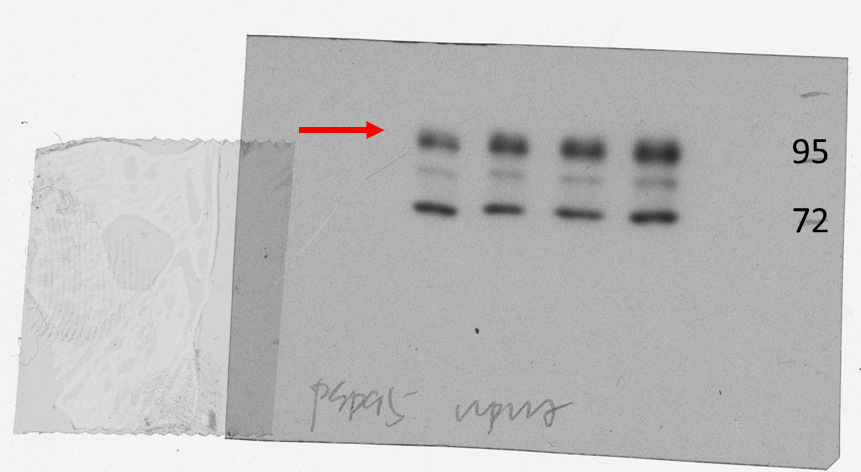


NOS1AP


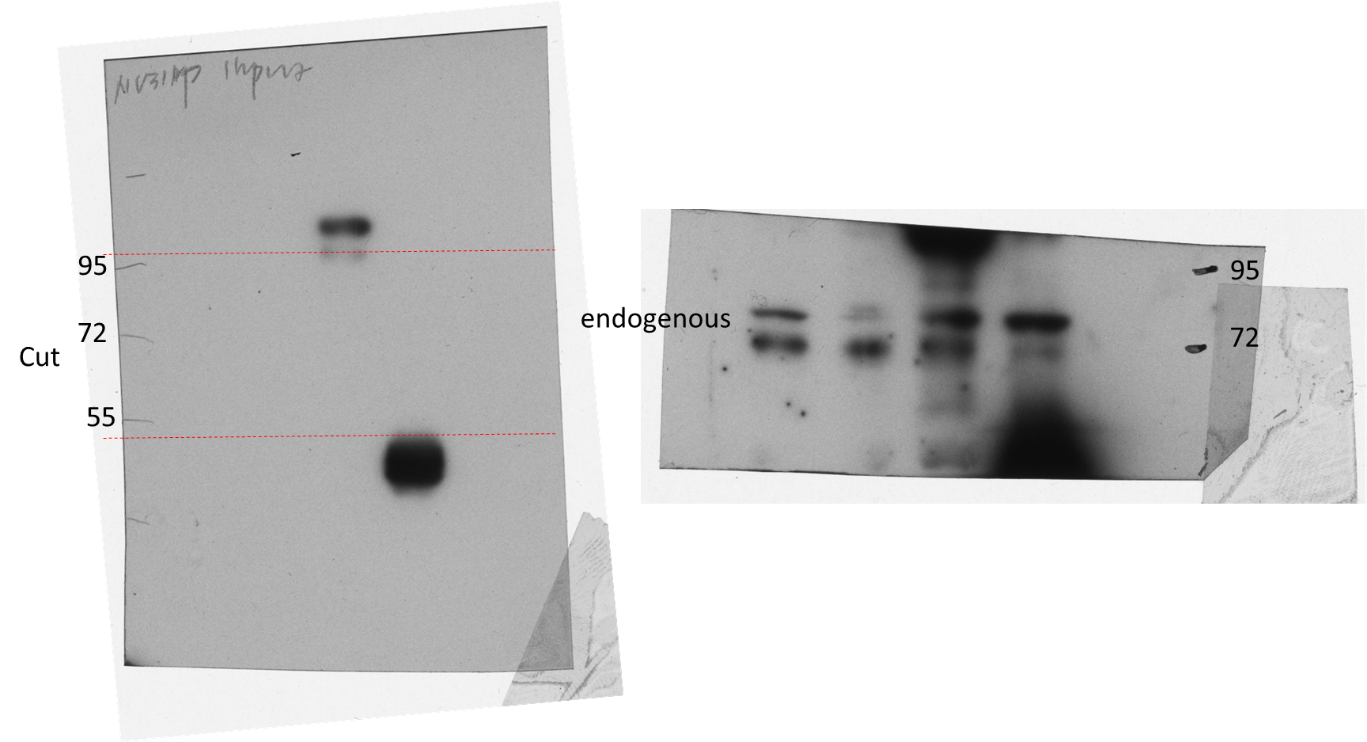


**Set2 Co-IP samples:**

nNOS


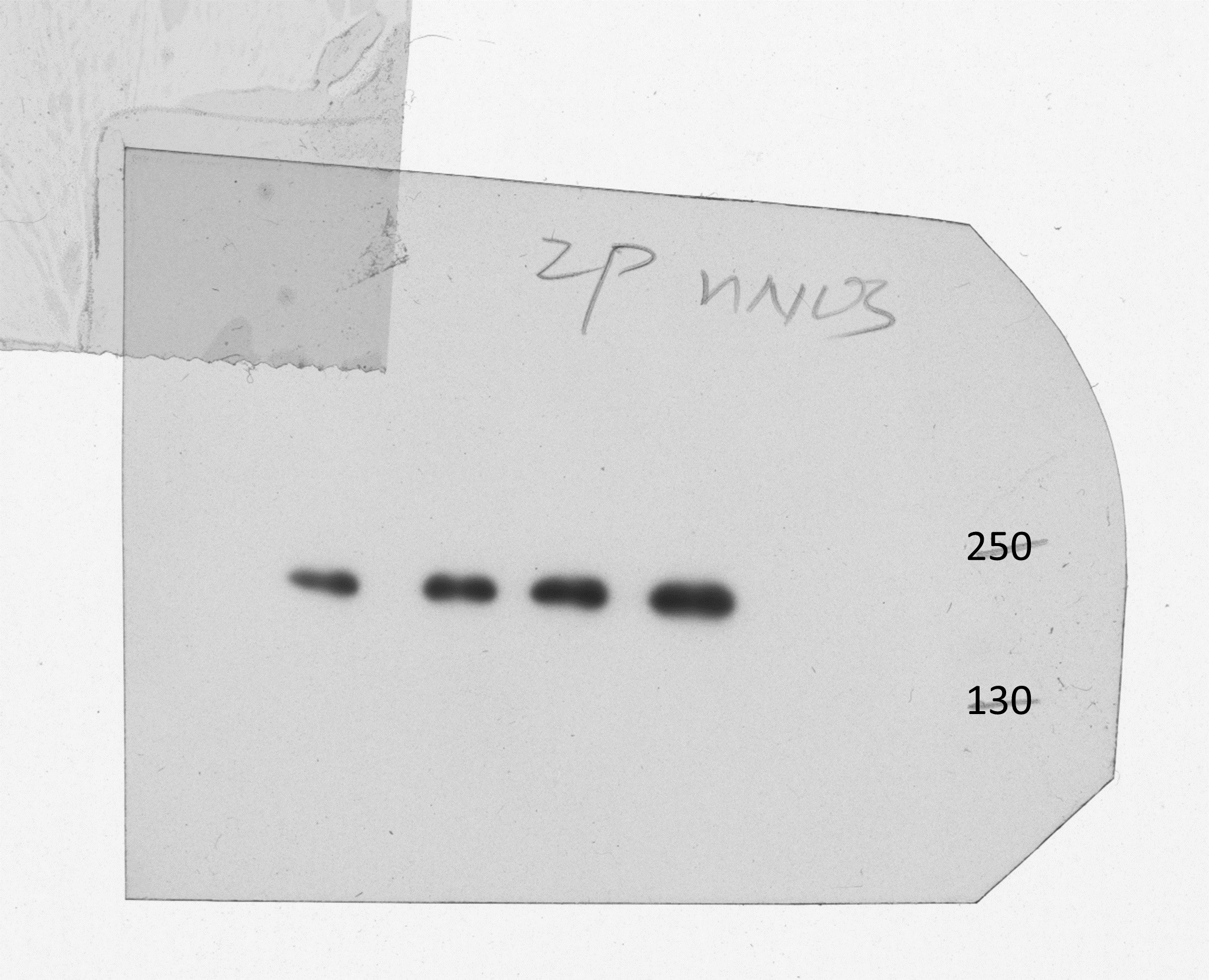


PSD95


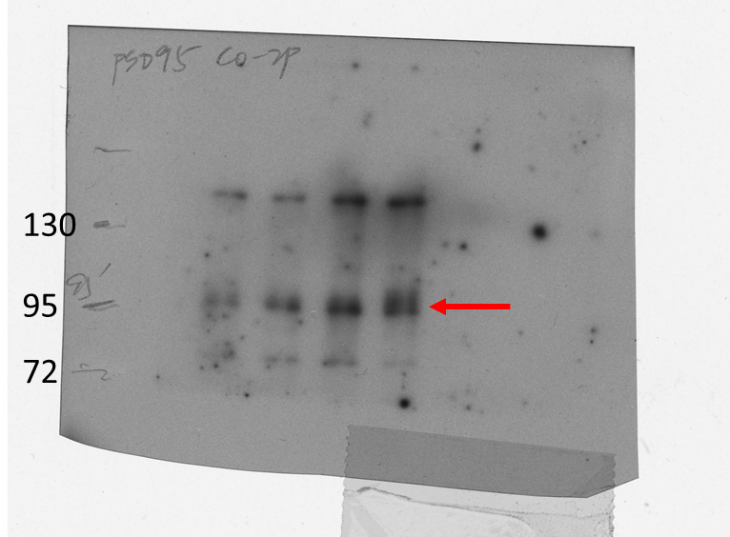


NOS1AP


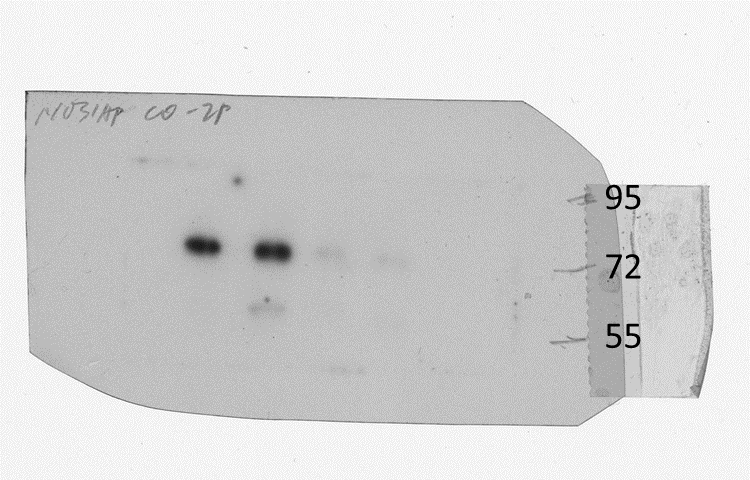


**Set 3 Input samples:**

nNOS


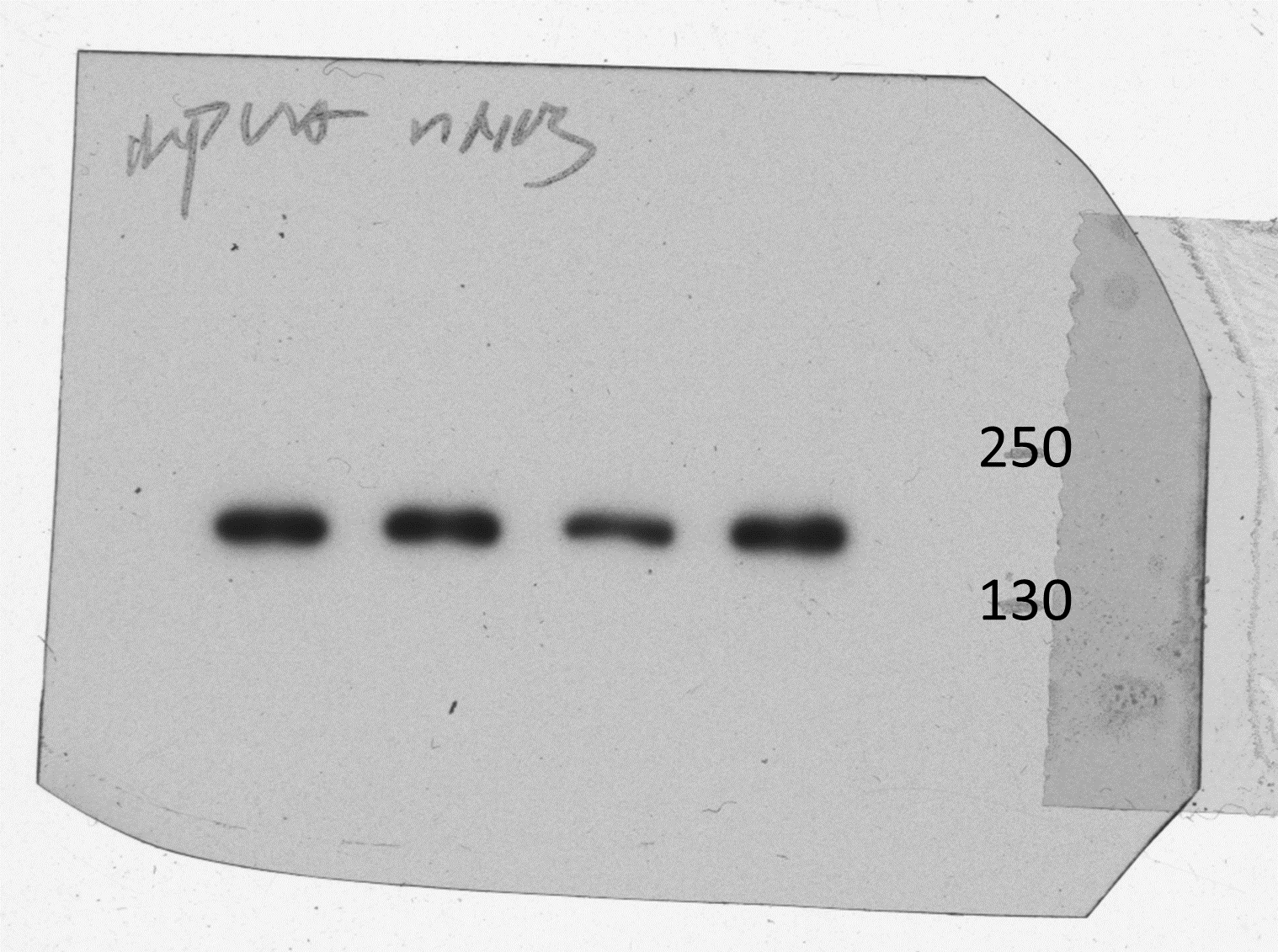


PSD95


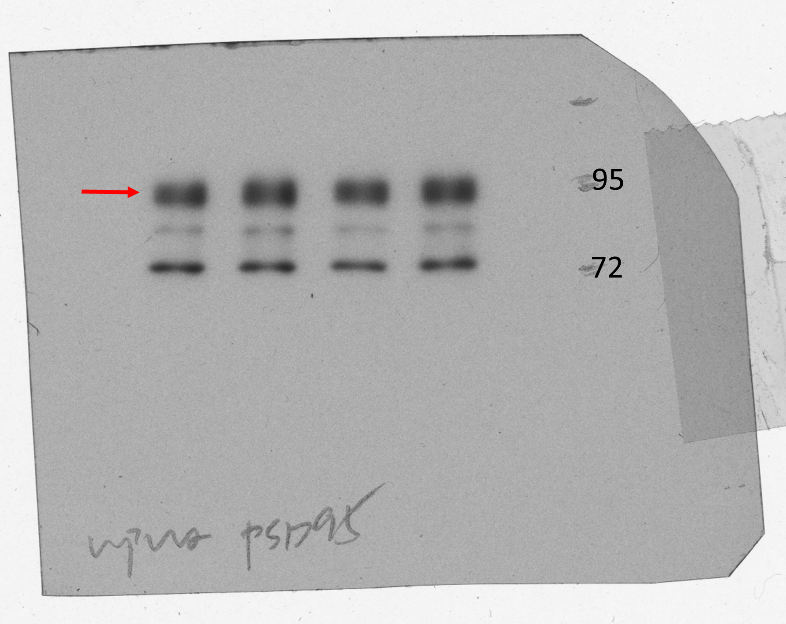


NOS1AP


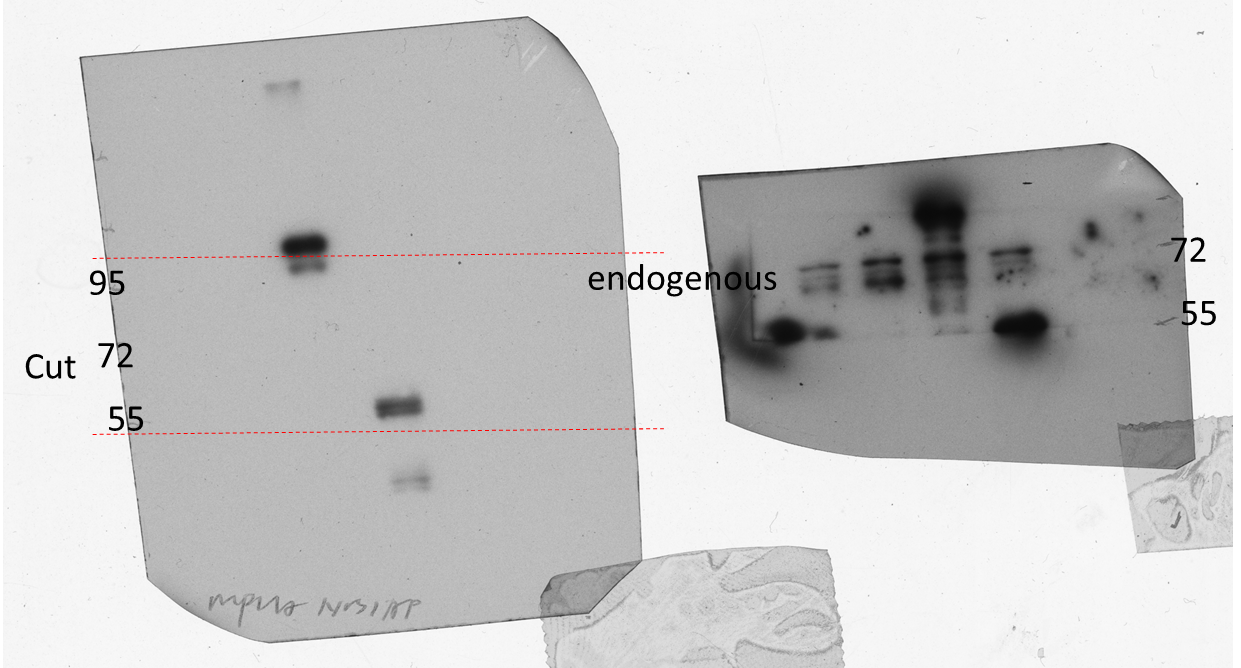


**Set3 Co-IP samples:**

nNOS


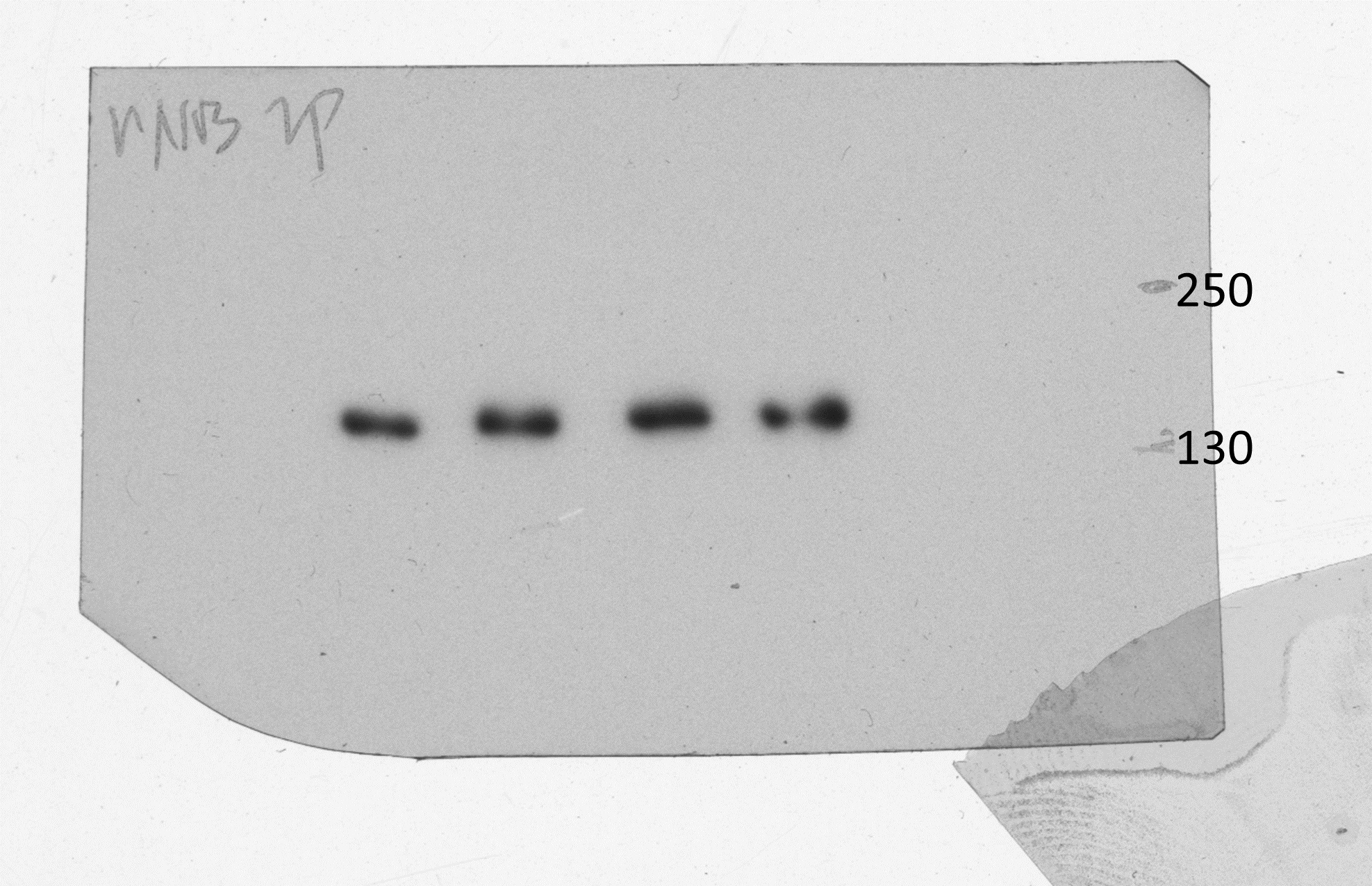


PSD95


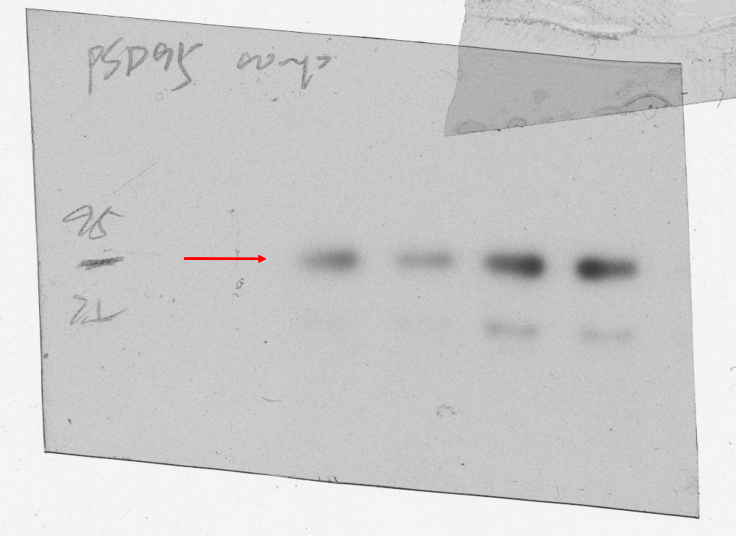


NOS1AP


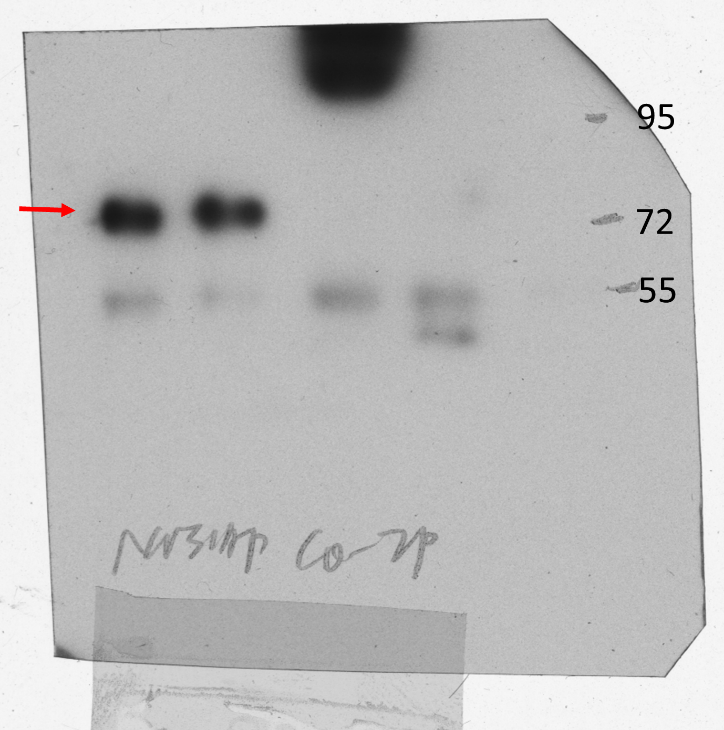

Supplement: Supplementary file 1 [file mmc1.docx]
