# Supplementary material for: Hippocampal overexpression of NOS1AP promotes endophenotypes related to mental disorders
Source: eBioMedicine. 2021 Aug 27;71:103565. doi: 10.1016/j.ebiom.2021.103565 (PMC8403735; doi:10.1016/j.ebiom.2021.103565)
Supplement: Supplementary file 2 [file mmc2.docx]

**Table S1: Sequences of oligonucleotides used in this study**

| **Name** | **Sequence (5’ 🡪 3’)** |
| --- | --- |
| WPRE-F | TGCTTCCCGTATGGCTTTCAT |
| WPRE-R | CATAGCGTAAAAGGAGCAACA |
| NOS1AP_396-503_-F | AGTgctagctCACTCACCACTGCTGGGCGC |
| NOS1AP_396-503_-R | GTCaagcttaCTACACGGCGATCTCATCATC |
