# Supplementary material for: Hippocampal overexpression of NOS1AP promotes endophenotypes related to mental disorders
Source: eBioMedicine. 2021 Aug 27;71:103565. doi: 10.1016/j.ebiom.2021.103565 (PMC8403735; doi:10.1016/j.ebiom.2021.103565)
Supplement: Supplementary file 3 [file mmc3.docx]

**Table S2: Primer pairs used for qPCR**

| **Name (Target)^a^** | **Sequences^b^** | **Target transcripts^c^** | **Std. curve^d^** |
| --- | --- | --- | --- |
| ***Actb*** (Mus musculus actin, beta) | F: GCTTCTTTGCAGCTCCTTCG  R: ATGCCGGAGCCGTTGTC | 60-156 of NM_007393.5 | y = -3·45x+18·4  r^2^ = 0·995  E = 0·951 |
| ***B2m*** (Mus musculus beta-2 microglobulin | F: ACTGACCGGCCTGTATGCTA  R: CAATGTGAGGCGGGTGGAA | 93-217 of NM_009735.3 | y = -2·97x+17·8  r^2^ = 0·956  E = 1·172 |
| ***Cpe*** (Mus musculus carboxypeptidase E) | F: AGGGTTTGTCCGTGACCTTC  R: GGAGCGGAGGCTGTAAGTTT | 1291-1437 of NM_013494.4 | y = -3·13x+14·9  r^2^ = 0·977  E = 1·088 |
| ***Dlg1*** (Mus musculus discs large MAGUK scaffold protein 1) | F: GTCACCAGCCAGGTACTCAC  R: AGTCCTGTTGAGCCACGATG | 1744-1845 of NM_007862.3, 1645-1746 of NM_001252433.1, NM_001252434.1, NM_001357282.1, 1403-1504 of NM_001252435.1, 1510-1611 of NM_001252436.1, 1498-1599 of NM_001252436.1, 1455-1556 of NM_001357281.1 | y = -3·6x+21·3  r^2^ = 0·984  E = 0·896 |
| ***Dlg3*** (Mus musculus discs large MAGUK scaffold protein 3) | F: GCAGCATGAGTTCTGGGTCT  R: GCTGTCACGAGTCCGATCAT | 1864-1958 of NM_016747.4 and NM_001177778.2, 765-859 of NM_001177779.1, 157-251 of NM_001177780.1, 1810-1904 of NM_001290402.1 | y = -2·71x+22·5  r^2^ = 0·978  E = 1·34 |
| ***Dlg4*** (Mus musculus discs large MAGUK scaffold protein 4) | F: GCTATGCTCCCCCAGACATC  R: GGTAGTCGGTGCCCAAGTAG | 1076-1162 of NM_007864.3, 1067-1153 of NM_001109752.1, 864-950 of NM_001370671.1, 862-948 of NM_001370672.1, 697-783 of NM_001370674.1, 893-979 of NM_001370675.1 | y = -3·26x+18·6  r^2^ = 0·986  E = 1·025 |
| ***Gria1*** (Mus musculus glutamate receptor, ionotropic, AMPA1 (alpha 1)) | F: ATCCTGACACAAAGGCCTGG  R: TCCTCCCGGACCAAGGTTAT | 1819-1918 of NM_001113325.2 and NM_008165.4, 1446-1545 of NM_001252403.1 | y = -3·52x+18·5  r^2^ = 0·964  E = 0·924 |
| ***Gria2*** (Mus musculus glutamate receptor, ionotropic, AMPA2 (alpha 2)) | F: ATGCGACCTGACCTCAAAGG  R: AGCAGAATCCAGCACAGCTT | 691-813 of NM_001083806.3, NM_013540.4, NM_001039195.3, NM_001357924.2 and NM_001357927.2 | y = -3·54x+16·6  r^2^ = 0·975  E = 0·916 |
| ***Grin2a*** (Mus musculus glutamate receptor, ionotropic, NMDA2A (epsilon 1)) | F: CTGCCGCATCTTCCATGTTG  R: TCCTTGCCATCCCAAGTCAC | 2102-2240 of NM_008170.4 | y = -3·52x+21  r^2^ = 0·955  E = 0·923 |
| ***Grin2b*** (Mus musculus glutamate receptor, ionotropic, NMDA2B (epsilon 2)) | F: TTCTGTCCCTTTATCCTCCGTCTT  R: TCATCTTCAGCTCGTCGACTC | 657-801 of NM_008171.4, 491-635 of NM_001363750.1 | y = -3·48x+19·2  r^2^ = 0·998  E = 0·939 |
| ***Hprt*** (Mus musculus hypoxanthine guanine phosphoribosyl transferase) | F: TGCTGACCTGCTGGATTACA  R: TTTATGTCCCCCGTTGACTGA | 371-490 of NM_013556.2 | y = -3·28x+18·8  r^2^ = 0·98  E = 1·016 |
| ***Gucy1a1*** (Mus musculus guanylate cyclase 1, soluble, alpha 1) | F: AAGCCGCAACAGAGTCTACC  R: AAGTGCAAGGTTCAGTCGCT | 571-658 of NM_021896.6, 684-771 of NM_001356988.1, 558-645 of NM_001356987.1 | y = -3·83x+22·7  r^2^ = 0·988  E = 0·824 |
| ***Guc1a2*** (Mus musculus guanylate cyclase 1, soluble, alpha 2) | F: GCCACCTTTGATAGAGTCTTGC  R: ATGGCACTTGATTCTGGGACA | 1565-1704 of NM_001033322.2 | y = -3·43x+21·8  r^2^ = 0·973  E = 0·956 |
| ***Gucy1b1*** (Mus musculus guanylate cyclase 1, soluble, beta 1) | F: TCTGCCAGGAGTCTGGCTAT  R: TAAATGGTGGCGAGGTGGTC | 367-472 of NM_017469.4 and NM_001161796.1 | y = -4·06x+21·8  r^2^ = 0·996  E = 0·764 |
| ***Map2k3*** (Mus musculus mitogen-activated protein kinase kinase 3) | F: GGACCTAGACATCAACATGCG  R: CATGCAGATCCATACATCCCC | 496-589 of NM_008928.5 | y = -3·17x+25·7  r^2^ = 0·965  E = 1·068 |
| ***Mapk14*** (Mus musculus mitogen-activated protein kinase 14) | F: TCACGCCAAAAGGACCTACC  R: ATTCCTCCAGTGACCTTGCG | 587-693 of NM_011951.3 and NM_001168508.1, 188-294 of NM_001168513.1 and NM_001357724.1, 632-738 of NM_001168514.1 | y = -4·07x+19·2  r^2^ = 0·996  E = 0·761 |
| ***Nos1ap*** (Mus musculus nitric oxide synthase 1 (neuronal) adaptor protein) | F: TCATGGTCTCTGTGGACGGT  R: TCTTGCTCTCATCCCACGTC | 706-786 of NM_001109985.2 | y = -3·54x+14·4  r^2^ = 0·98  E = 0·915 |
| ***Nos1*** (Mus musculus nitric oxide synthase 1, neuronal) | F: CTGGTGAAGGAACGGGTCAG  R: CCGATCATTGACGGCGAGAAT | 469-588 of NM_008712.3 | y = -3·78x+16·1  r^2^ = 0·999  E = 0·838 |
| ***Rasd1*** (Mus musculus RAS, dexamethasone-induced 1) | F: GATGTGCCCAAGCGACTCT  R: TGAGGAAGCGCGACACAAT | 166-275 of NM_009026.5 | y = -3·81x+22  r^2^ = 0·99  E = 0·831 |
| ***Scrib*** (Mus musculus scribbled planar cell polarity) | F: GGTTTTGACACCAGCACCAC  R: GTTCCCGGTCAATGGACGAA | 3926-4046 of NM_134089.2, NM_001310542.1 and NM_001310543.1 | y = -3·28x+24·2  r^2^ = 0·976  E = 1·016 |
| ***Sdha*** (Mus musculus succinate dehydrogenase complex, subunit A, flavoprotein (Fp)) | F: GGACAGGCCACTCACTCTTAC  R: CACAGTGCAATGACACCACG | 616-745 of NM_023281.1 | y = -3·92x+19·2  r^2^ = 0·991  E = 0·799 |
| ***Syn1*** (Mus musculus synapsin I) | F: TGCCCAGATGGTTCGACTAC  R: CACAGGGTATGTTGTGCTGC | 816-927 of NM_013680.4 and NM_001110780.1 | y = -3·67x+20·6  r^2^ = 0·994  E = 0·874 |
