# Supplementary figures and images for: Hippocampal overexpression of NOS1AP promotes endophenotypes related to mental disorders
Source: eBioMedicine. 2021 Aug 27;71:103565. doi: 10.1016/j.ebiom.2021.103565 (PMC8403735; doi:10.1016/j.ebiom.2021.103565)

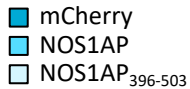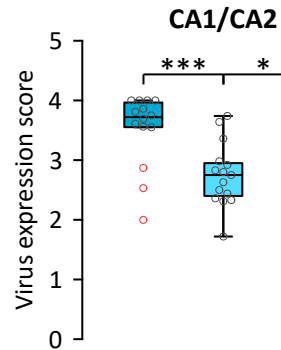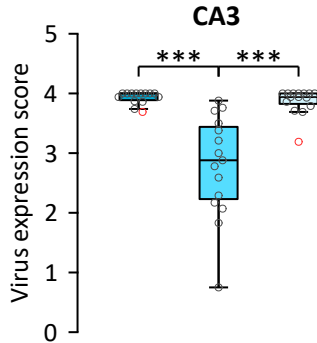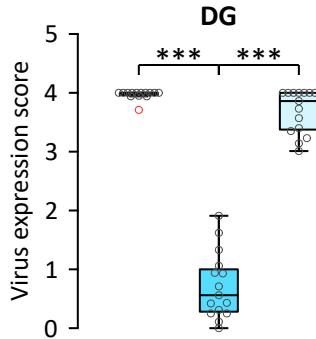

Supplement: Supplementary file 4 [file mmc4.pdf]

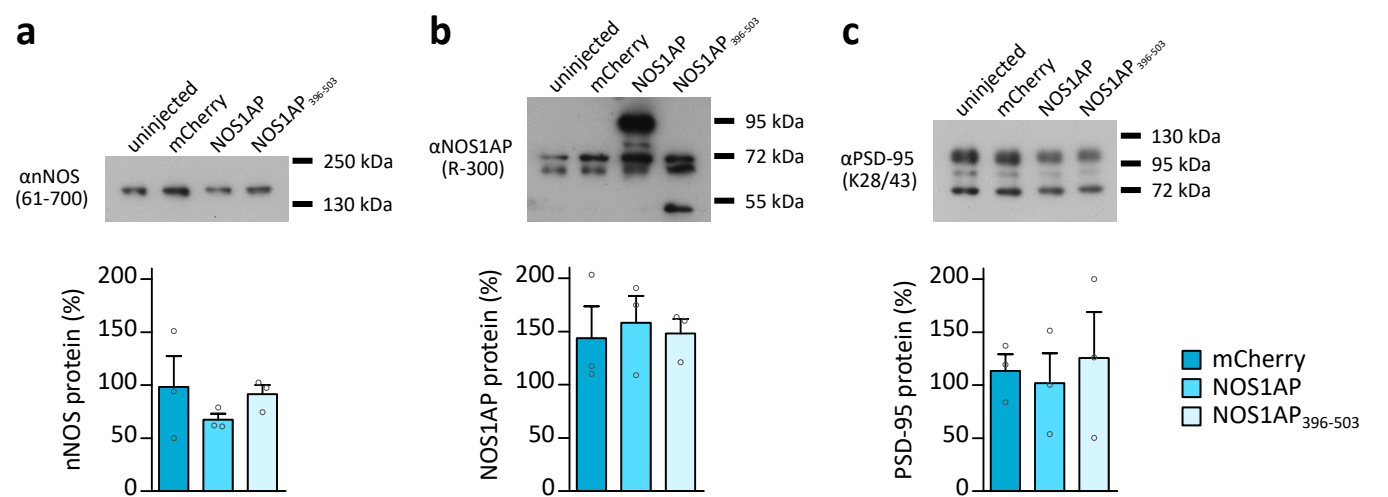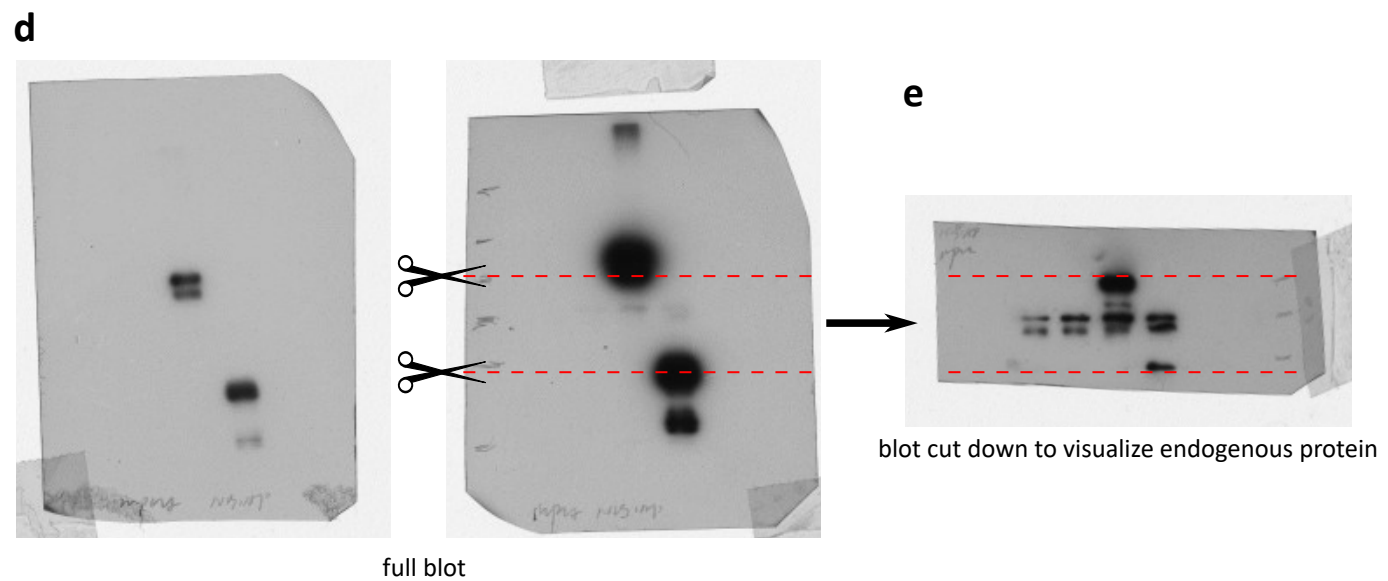

Supplement: Supplementary file 5 [file mmc5.pdf]

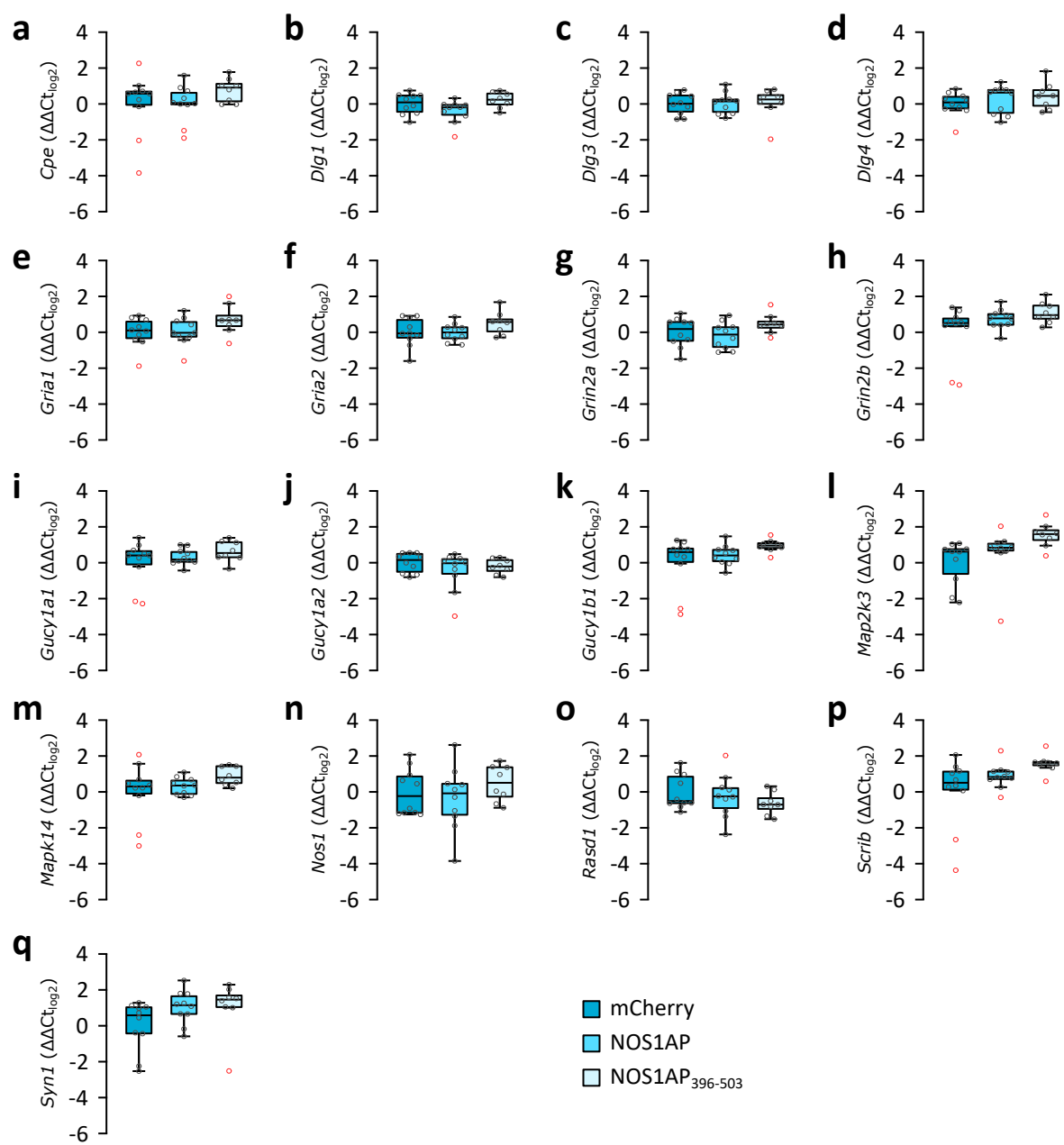

Supplement: Supplementary file 6 [file mmc6.pdf]
